# Signaling by intracellular β_2_-adrenergic receptors regulates AMPA receptor trafficking and synaptic plasticity

**DOI:** 10.1016/j.celrep.2025.116011

**Published:** 2025-07-17

**Authors:** Boram Lee, Xiaomin Xing, Erik A. Hammes, Zhuoer Zeng, Zoila M. Estrada-Tobar, Karam Kim, Kyle E. Ireton, Kwun Nok Mimi Man, Ariel A. Jacobi, Ruben A. Berumen, Justin C. Weiner, Ao Shen, Bing Xu, Joanne Wang, Paul J. Gasser, Manuel F. Navedo, Yang K. Xiang, Elva Díaz, Chao-Yin Chen, Mary C. Horne, Johannes W. Hell

**Affiliations:** 1 Department of Pharmacology, University of California, Davis, Davis, CA 95616-8636, USA; 2 VA Northern California Health Care System, Mather, CA 95655, USA; 3 Department of Pharmaceutics, University of Washington, Seattle, WA 98195-7280, USA; 4 Department of Biomedical Sciences, Marquette University, Milwaukee, WI 53201-1881, USA; 5 Present address: Samsung Bioepis, Incheon, South Korea; 6 Present address: Shenzhen Bay Laboratory, Shenzhen 518132, China; 7 Present address: Arrhythmia Technologies Institute, Greenville, SC 29615, USA; 8 Present address: Genentech, South San Francisco, CA 94080, USA; 9 Present address: Syneos Health, South San Francisco, CA 94080, USA; 10 Present address: Max-Planck-Institute, Jupiter, FL 33458, USA; 11 Present address: Guangzhou Medical University, Guangzhou 511436, China; 12 These authors contributed equally; 13 These authors contributed equally; 14 Lead contact

## Abstract

Signaling by norepinephrine (NE) via adrenergic receptors (ARs) mediates attention, yet the underlying molecular mechanisms are largely unknown. AMPA receptors (AMPARs) form a complex with β_2_ARs, G_s_, adenylyl cyclase, and protein kinase A (PKA) to augment AMPAR phosphorylation and, thereby, surface expression. We show that signaling by intracellular β_2_ARs is required for these effects and two different forms of long-term potentiation (LTP) that depend on β_2_AR signaling and phosphorylation of the AMPAR GluA1 subunit on S845. Inhibition of two NE transporters, the organic cation transporter 3 (OCT3) and the plasma membrane monoamine transporter (PMAT), impairs phosphorylation of the AMPAR GluA1 subunit on S845 by PKA, GluA1 surface insertion, both forms of LTP, and upregulation of miniature excitatory postsynaptic currents upon injection of NE into neurons. These results provide strong evidence for signaling by NE upon its transport into neurons in general and specifically in synaptic plasticity.

## In brief

Lee et al. show that OCT3 and PMAT transport norepinephrine (NE) into neurons to stimulate β_2_ARs that are in their interior. The ensuing phosphorylation of GluA1 on S845 by PKA drives AMPARs to the surface and postsynaptic sites during PKA-dependent forms of LTP.

## Graphical Abstract

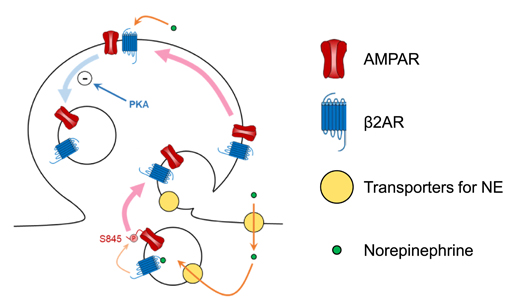

## INTRODUCTION

Norepinephrine (NE) is widely released throughout the brain to support arousal and learning.^1–8^ NE acts via α- and β-adrenergic receptors (ARs). The β_2_AR forms a unique signaling complex with the AMPA receptor (AMPAR),^9–11^ which also contains G_s_, adenylyl cyclase, protein kinase A (PKA), and the counteracting phosphatase PP2B.^10,12–17^ The formation of this signaling complex suggests that the AMPAR is an important target for NE signaling.

S845 in the AMPAR GluA1 subunit is the main phosphorylation site for PKA in AMPARs in adult rodents.^18–20^ This phosphorylation is important for activity-driven surface insertion and, ultimately, postsynaptic accumulation of GluA1^9,21–26^ during various forms of synaptic plasticity.^20,27–32^ Surface insertion of AMPARs, including newly synthesized receptors, occurs through recycling endosomes (REs)^33^ at perisynaptic sites.^27,34–36^ Upon insertion, AMPARs can readily diffuse to postsynaptic sites where their confinement is increased during long-term potentiation (LTP)^37–39^ (Figure S1A). By stimulating S845 phosphorylation, βAR signaling promotes different kinds of LTP in the hippocampus,^25,40–43^ including LTP induced by a prolonged (90–180 s) theta oscillation (5–12 Hz) tetanus paired with the application of a βAR agonist^7,40,41,44^ (see also Birnie et al.^45^), which we call “prolonged theta-tetanus LTP” (PTT-LTP). In fact, PTT-LTP strictly requires signaling by the β_2_AR as well as S845 phosphorylation.^32^

Remarkably, only GluA1 subunits that are part of the β_2_AR-AMPAR complex become phosphorylated on S845 upon βAR stimulation.^9,46^ Restriction of cyclic AMP (cAMP) signaling to individual β_2_ARs with an effective radius of ∼60 nm has recently been directly observed by microscopic cAMP imaging with “nanorulers.”^47,48^ Furthermore, acute displacement of the β_2_AR from AMPARs with specific peptides blocks the increase in phosphorylation of GluA1 on S845, GluA1 surface expression, and amplitude of AMPAR-mediated excitatory postsynaptic currents (EPSCs) induced via β_2_AR signaling.^9^ Critically, the increase in EPSC amplitude was also blocked by inhibitors of exocytosis.^9^ If, as assumed until recently, NE acted exclusively on ARs at the plasma membrane, these findings created the following conundrum: how can *extracellular* NE mobilize *intracellular* AMPARs by inducing their phosphorylation of GluA1 on S845 to upregulate their surface insertion and EPSC size, given that S845 phosphorylation is limited to β_2_AR-associated AMPARs? In other words, how can extracellular NE engage signaling that is spatially restricted to individual β_2_AR-AMPAR complexes that reside inside neurons to initiate their trafficking to the cell surface?

It has been known for a while that cAMP synthesis inside cells can be mediated following endocytosis of the thyroid-stimulating hormone receptor, the parathyroid hormone receptor, and the vasopressin receptor with their cognate agonists.^49–51^ However, recent work indicates that β_2_ARs and β_1_ARs that are localized in the cell interior can undergo activation to mediate intracellular signaling.^52,53^ Subsequent work suggests that the membrane-impermeant cognate βAR ligand epinephrine is transported by the cation transporter organic cation transporter 3 (OCT3), which mediates the uptake of the positively charged monoamines, including NE,^54,55^ through the plasma membrane and then presumably into the lumen of the Golgi apparatus to activate β_1_ARs in this compartment.^56^ This signaling is independent of endocytosis of β_1_AR at the cell surface.^56^ It was impaired by low temperature, which would inhibit transport activity but not diffusion, and by corticosterone (CORT), an inhibitor of OCT3, but not by the inhibition of endocytosis of β_1_AR with Dyngo-4a. Analogous results were obtained for dopamine uptake and intracellular activation of the dopaminergic D_1_ receptor.^57^ Thus, we hypothesized that signaling by NE occurs inside neurons and that NE is transported into neurons to stimulate GluA1-associated β_2_ARs and, thereby, phosphorylation of these GluA1 subunits inside cells, which then promotes surface expression of those phosphorylated GluA1-containing AMPARs.

The transporters OCT1, OCT2, OCT3, and plasma membrane monoamine transporter (PMAT) constitute the so-called uptake 2 system for monoamines.^54,55,58^ In contrast to uptake 1 (which exhibits high affinity but low capacity transport), uptake 2 is characterized by a high transport capacity. Uptake 1 includes the NE transporter NET and the dopamine transporter DAT, which perform presynaptic NE and dopamine reuptake, respectively. OCT3, as well as OCT2 and PMAT, is strongly expressed in neurons.^58–61^ In support of the functional relevance of these transporters in the brain, knockout (KO) mice of each of these individual transporters show clear alterations in their behavior, including changes in anxiety.^60–62^

Here, we provide evidence that indicates that NE is transported into neurons to stimulate the phosphorylation of intracellular AMPARs. Our extensive in-depth analysis of the effects of pharmacological inhibition of OCT3 and PMAT and genetic loss of OCT3 and PMAT in KO mice indicate that NE transport into intact neurons mediates S845 phosphorylation of GluA1 subunits that are inside cells, GluA1 surface expression, and two different forms of LTP, PTT-LTP and LTP, induced by a single 1-s-long, 100-Hz tetanus (HFS-LTP) in young adult mice. These findings establish that NE can be transported into not only heterologous models like HEK293 and HeLa cells but also neurons to stimulate intracellular β_2_AR signaling *in situ*. Of immediate functional relevance is our finding that such intracellular signaling by NE is part of the molecular mechanisms that drive LTP.

## RESULTS

### Inhibitors of OCT3 and PMAT reduce NE-induced S845 phosphorylation

**Figure 1.**
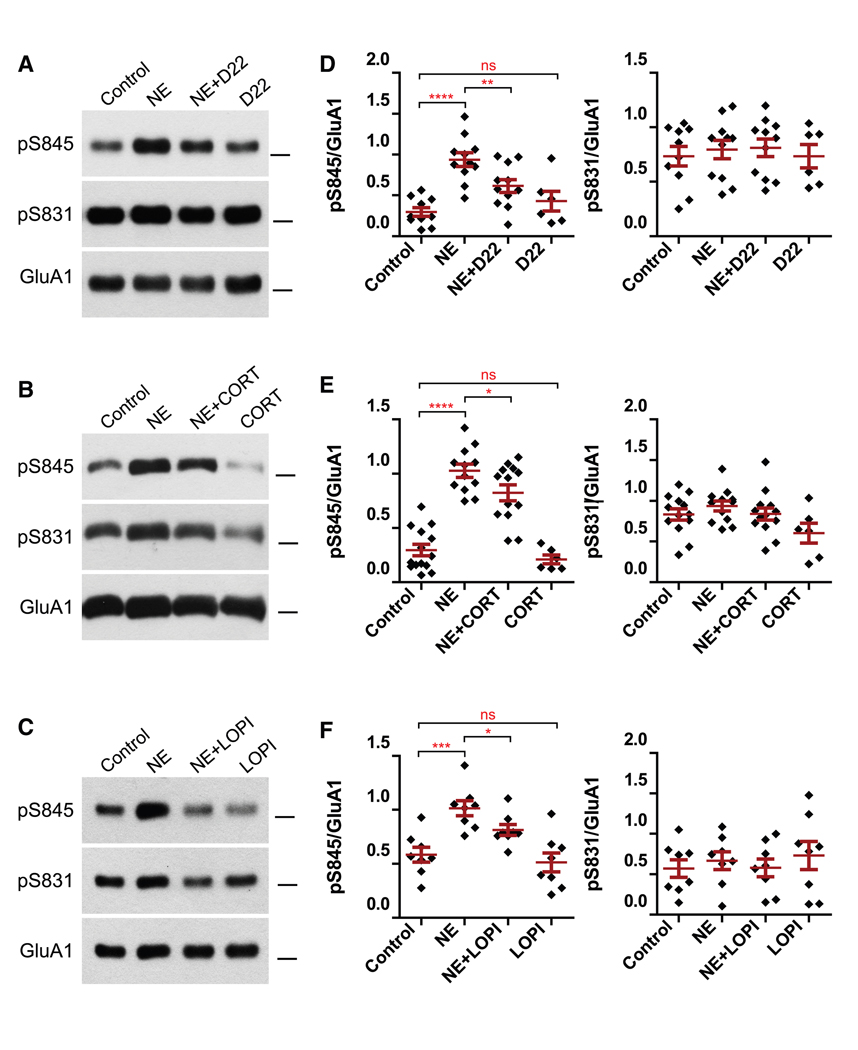
D22, CORT, and LOPI inhibit NE-induced phosphorylation of GluA1 at S845 (A–C) Forebrain slices from wild-type mice were pre-incubated with artificial cerebrospinal fluid (ACSF) for 5 min containing vehicle (DMSO), 100 nM D22 (A), 10 μM CORT (B), or 10 μM LOPI (C); incubated for 10 min with the respective inhibitor + 1 μM NE or vehicle (dimethylsulfoxide [DMSO]); and solubilized (1% Triton X-100) before ultracentrifugation, immunoprecipitation of GluA1, and sequential immunoblotting with antibodies against pS845, pS831, and the GluA1 C terminus (total GluA1). The bars on the right sides of each blot indicate the 100 kD marker position. (D–F) For quantification, signals for pS845 and pS831 were normalized to total GluA1 signals (D, *n* ≥ 6 from 6 mice; E, *n* ≥ 6 from 7 mice; F, *n* ≥ 8 from 3 mice; bars and whiskers represent means ± SEM; data were analyzed by one-way ANOVA and significance of differences between control versus NE, NE versus NE + inhibitor, and control versus inhibitor by Fisher’s least significant difference [LSD] post hoc test; *p < 0.05, **p < 0.01, ***p < 0.001, ****p < 0.0001, and ns, not significant). D22 inhibited NE-induced S845 phosphorylation by 50%, CORT by 28%, and LOPI 47%.

We first used decynium 22 (D22) to test whether transport of NE into neurons by the uptake 2 system induces NE signaling inside neurons because it is the most potent established inhibitor for uptake 2 transport (IC_50_ in native cells: 3–4 nM^63,64^) with strong selectivity for OCT3 and PMAT over OCT1 and OCT2.^65–69^ Forebrain slices of the dorsal hippocampus and surrounding cortical areas were incubated with NE in the presence or absence of D22. D22 reduced NE-induced S845 phosphorylation by 50% without affecting basal phosphorylation (Figures 1A and 1D). To differentiate between OCT3 and PMAT, we used CORT, which preferentially inhibits OCT3 (Figures 1B and 1E), and lopinavir (LOPI), which preferentially inhibits PMAT (Figures 1C and 1F).^68–70^ Like D22, these two inhibitors also significantly reduced NE-induced S845 phosphorylation by 28% and 47%, respectively. As expected, the inhibitors only partially blocked NE-induced S845 phosphorylation because they do not affect the stimulation of β_2_ARs by NE at the cell surface. Ca^2+^/calmodulin-dependent protein kinase (CaMKII) and PKC, but not PKA, can phosphorylate GluA1 on S831.^18,71^ Thus, S831 phosphorylation served as a control readout for assessing the potential engagement of other signaling pathways. NE did not stimulate S831 phosphorylation beyond baseline levels, and, as expected, none of the inhibitors had an effect here either. This finding is important because NE can activate not only βARs but also α1ARs to stimulate G_q_-phospholipase C (PLC)-inositol trisphosphate (IP3)/Ca^2+^-PKC signaling. However, this α1AR-PKC signaling pathway was clearly not engaged, as NE did not augment the phosphorylation of GluA1 on S831 by PKC.

The above results suggest that OCT3 or PMAT, or most likely both, transports NE into neurons and then into vesicles for signaling by β_2_ARs that are inside neurons, which typically couple to G_s_ but not G_q_. Of note, the NE transporter termed NET is thought to mediate the clearance of NE from the extracellular space to terminate NE signaling and reuptake for re-use in presynaptic terminals. However, the selective NET blocker desipramine (DESI; 1 μM) had no effect (Figures S1B and S1C), even though this concentration is orders of magnitude higher than the 4 nM IC_50_ for DESI.^72^ Thus, there was no significant contribution of NET in the regulation of S845 phosphorylation by signaling of NE when inside neurons.

### Inhibitors of OCT3 and PMAT block NE-induced GluA1 surface insertion

**Figure 2.**
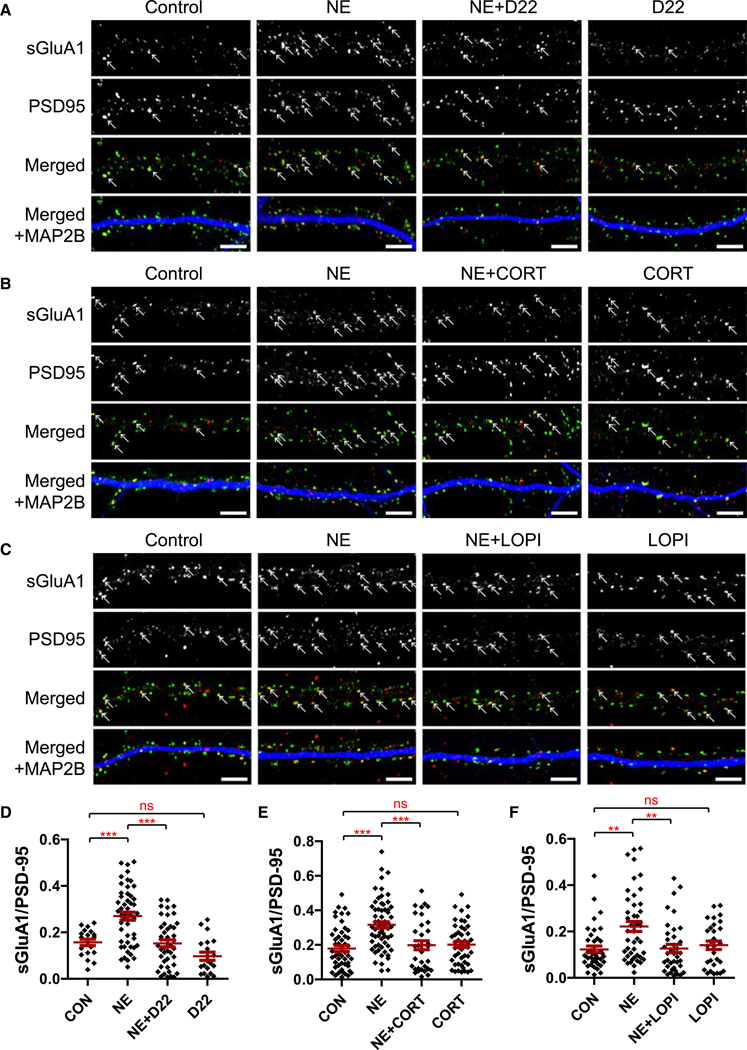
D22, CORT, and LOPI block NE-induced surface insertion of GluA1 (A–C) Confocal images of hippocampal cultures (days *in vitro* [DIV] 20–22) treated with vehicle (DMSO) or NE (1 μM) + 10 nM D22 (A), 10 μM CORT (B), or 10 μM LOPI (C) prior to fixation are as indicated. Images of surface GluA1 were obtained by incubating the fixed but intact (unpermeabilized) cells with primary antibodies against the extracellular N terminus of GluA1 (sGluA1; red in top and bottom rows). Intracellular staining of the postsynaptic marker PSD-95 (green in middle and bottom rows) and dendritic marker MAP2B (blue in bottom rows) of the same neurons were obtained by incubation with respective primary antibodies following permeabilization (scale bars, 5 μm). (D–F) Number of detectable sGluA1 puncta colocalized with PSD-95 puncta versus total number of PSD-95 puncta as a readout of relative sGluA1 content at or near postsynaptic sites (D, *n* ≥ 19 from 4 independent cultures; E, *n* ≥ 22 from 5 cultures; F, *n* ≥ 28 from 5 cultures; bars and whiskers represent means ± SEM; data were analyzed by one-way ANOVA and significance of differences between control versus NE, NE versus NE + inhibitor, and control versus inhibitor assessed by Fisher’s least significant difference [LSD] post hoc test, D and E, or Kruskal-Wallis test and Dunn’s multiple comparisons test, F [which was not normally distributed]; *p < 0.05, **p < 0.01, and ns, not significant). D22 inhibited NE-induced increase in sGluA1 density at (peri)synaptic sites by 104%, CORT by 86%, and LOPI 96%.

Phosphorylation of S845 by PKA is important for surface insertion and postsynaptic accumulation of GluA1.^9,21–26^ Furthermore, stimulation of GluA1-associated β_2_AR augments not only S845 phosphorylation but also GluA1 surface expression.^9,10^ We, therefore, tested whether stimulation of GluA1 surface expression by NE requires the uptake 2 system for intracellular NE signaling. Mature cultures of dissociated hippocampal neurons were treated with NE, D22, CORT, and LOPI before surface labeling with antibodies against the extracellular N terminus of GluA1 (Figure 2). Immunofluorescent GluA1 surface labeling (sGluA1) was quantified by determination of the number of sGluA1 puncta that overlapped with puncta for the postsynaptic marker PSD-95 and normalized to the total PSD-95 puncta number. This analysis reflects the prevalence of sGluA1 at both postsynaptic and perisynaptic sites, which cannot be easily discerned from one another by confocal microscopy. As we expected,^9^ NE application consistently more than doubled the number of sGluA1 puncta that colocalized with PSD-95. D22 completely blocked this increase by 104%, and CORT and LOPI strongly impaired it by 86% and 96%, respectively. Neither inhibitor significantly affected sGluA1 labeling by itself. That these inhibitors had much stronger effects on NE-induced surface localization than on S845 phosphorylation of GluA1 provided further support for the hypothesis that surface insertion is regulated by NE acting mostly inside neurons as opposed to S845 phosphorylation of the total pool of GluA1 with a substantial pool being already at the cell surface, whose phosphorylation can be induced by signaling via β_2_ARs that are at the surface, which is independent of NE transport and therefore insensitive to the blocking of NE transport.

### Inhibitors of OCT3 and PMAT impair PTT-LTP induced by NE but not by isoproterenol

**Figure 3.**
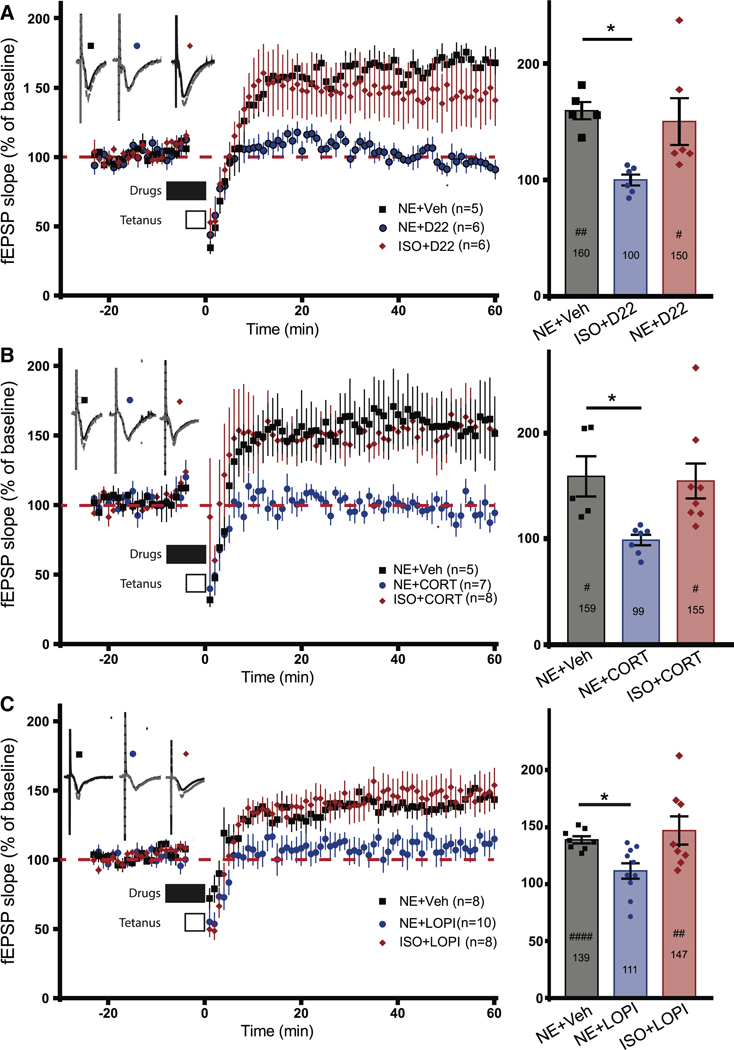
D22, CORT, or LOPI blocks PTT-LTP with NE but not ISO Field EPSPs (fEPSPs) were recorded from acute hippocampal slices before and after 5-Hz, 3-min tetani (PTT; open bars). Plots show the time course of the initial slopes. Solid black horizontal bars indicate the time of co-perfusion with 1 μM NE and vehicle (Veh; 0.01% DMSO), 1 μM NE + transport inhibitor (in DMSO), or 1 μM ISO + transport inhibitor. Top left insets: sample traces of fEPSP recordings 20 min prior to tetanus (solid lines) and 30 min post-tetanus (dashed lines). Right insets: bar graphs of potentiation of fEPSPs 45–50 min after PTT (gray: NE; blue: NE + inhibitors; red: ISO + inhibitors). Shown are means ± SEM. The exact value of the level of fEPSP after potentiation is given inside the bars. Paired t test was used to determine significant differences of fEPSP slope averages from 5 to 10 min prior to drug treatment and 45–50 min post-PTT (^#^*p* < 0.05, ^##^*p* < 0.01, and ^####^*p* < 0.0001). One-way ANOVA was used to determine differences between treatment groups with Dunnett’s multiple comparisons test to determine significance between control (NE + Veh) and the other treatment groups, *p < 0.05. (A) D22 (1 μM) blocked induction of PTT-LTP with NE but not ISO. (B) CORT (1 μM) blocked induction of PTT-LTP with NE but not ISO. (C) LOPI (10 μM) blocked induction of PTT-LTP with NE but not ISO.

PTT-LTP is induced by a 90–180 s tetanus of 5–10 Hz when paired with βAR stimulation.^32,40,73^ Phosphorylation of S845 by PKA is required for PTT-LTP^32^ likely because it fosters perisynaptic surface insertion of AMPARs and thereby their availability for postsynaptic accumulation.^9,21–23,25–27^ To advance our mechanistic insight into PTT-LTP, as well as to explore the physiological relevance of the intracellular NE signaling implicated by our above findings, we tested whether impairing OCT3 and PMAT activity would affect the PTT-LTP induced by NE. Application of either NE or isoproterenol (ISO) induced, on average, an ∼10% increase in postsynaptic strength that did not reach statistical significance versus the baseline present before drug application (Figure S2A). However, pairing NE or ISO application with a 3-min, 5-Hz tetanus resulted in robust PTT-LTP, reflected by an ∼42% increase in field excitatory postsynaptic potential (fEPSP) slope 45–50 min post-tetanus versus that seen 5–10 min pre-tetanus (Figure S2A). Importantly, this PTT-LTP induced by NE pairing was completely blocked by CORT and D22 and strongly reduced by LOPI (Figure 3). It is unlikely that CORT could have acted via canonical nuclear receptor signaling, as the incubation time lasted only 10 min. However, to exclude other side effects that would impair mechanisms required for PTT-LTP, we repeated these experiments with ISO. In contrast to NE, ISO is thought to be membrane permeant due to its considerably higher lipophilicity. Calculated logP values for ISO vary between −0.27 and +0.24, and the experimentally determined logP value is +1.4 (Toxin and Toxin Target Database: http://www.t3db.ca/toxins/T3D4979), with more positive values indicating higher lipophilicity. Compounds with logP > −0.5 are considered to be typically able to permeate biological membranes and efficiently reach intracellular compartments.^74,75^ We found that in the presence of each of the three transport inhibitors, ISO supported full, unimpaired induction of PTT-LTP, while NE did not in the interleaved experiments (Figure 3). These results clearly show that the OCT3 and PMAT inhibitors impaired PTT-LTP because they affected the intracellular access of NE and not because they affected other molecular mechanisms that were also required for PTT-LTP.

DESI, which blocks NET, did not affect PTT-LTP at 1 μM (Figure S2B). These results argue against a significant contribution by NET in PTT-LTP. However, we cannot exclude that NET mediates intracellular signaling by NE in other functions, especially at presynaptic sites of monoaminergic synapses.

### Deletion of OCT3 and PMAT inhibits PTT-LTP induced by NE but not by ISO

**Figure 4.**
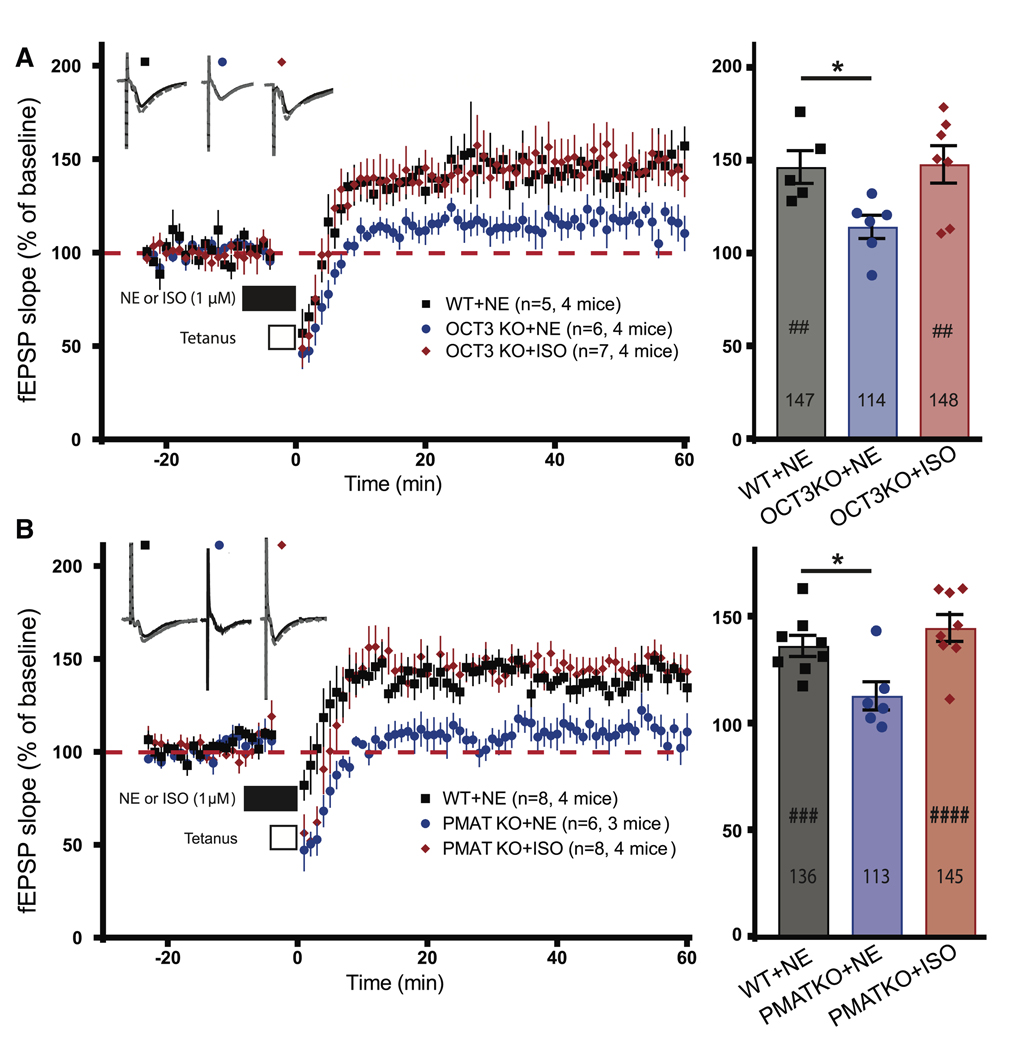
OCT3 and PMAT KO mice show impaired PTT-LTP with NE but not with ISO Field EPSPs (fEPSPs) were recorded from acute hippocampal slices from wild-type (WT) and KO mice before and after 5-Hz, 3-min tetani (PTT; open bar). Plots show the time course of the initial slopes. Solid horizontal black bars indicate the time of perfusion with vehicle (H_2_O), 1 μM NE (in H_2_O), or 1 μM ISO (in H_2_O). Top left insets: sample traces of fEPSP recordings 20 min prior to tetanus (solid lines) and 30 min post-tetanus (dashed lines). Right insets: bar graph of potentiation of fEPSPs 45–50 min after PTT (gray: WT with NE; blue: KO with NE; red: KO with ISO). Shown are means ± SEM. The exact value of the level of fEPSP after potentiation is given inside bars. Paired t test was used to determine significant differences of fEPSP slope averages from 5 to 10 min prior to drug treatment and 45–50 min post-PTT (^##^*p* < 0.01, ^###^*p* < 0.001, and ^####^*p* < 0.0001). One-way ANOVA was used to determine differences between treatment groups with Dunnett’s multiple comparisons test to determine significance between control (NE + Veh) and the other treatment groups, *p < 0.05. (A) In OCT3 KO slices, full PTT-LTP was induced with ISO but not NE. (B) In PMAT KO slices, full PTT-LTP was induced with ISO but not NE.

We used OCT3 and PMAT KO mice to rule out the side effects of OCT3 and PMAT inhibitors and determine whether OCT3, PMAT, or both are required for PTT-LTP. Basal synaptic transmission was unaltered in the two KO mice with respect to paired-pulse facilitation and the excitatory input-output relationships (IORs) for both AMPAR- and NMDA receptor (NMDAR)-mediated fEPSPs, indicative of normal presynaptic glutamate release and postsynaptic AMPAR and NMDAR responses, respectively (Figure S3). Consistent with the results with the transport inhibitors, PTT-LTP was impaired in both OCT3 and PMAT KO mice when induced with NE but not with ISO (Figures 4A and 4B). These results disambiguate the roles of OCT3 and PMAT and clearly show that both transports are required for full PTT-LTP induced with NE. However, some residual potentiation appears to be present in each KO mouse line, although this residual potentiation was statistically not significantly different from non-potentiated baseline responses.

### Abrogation of S845 phosphorylation and of β_2_AR stimulation inside neurons impairs a PKA-dependent form of HFS-LTP

**Figure 5.**
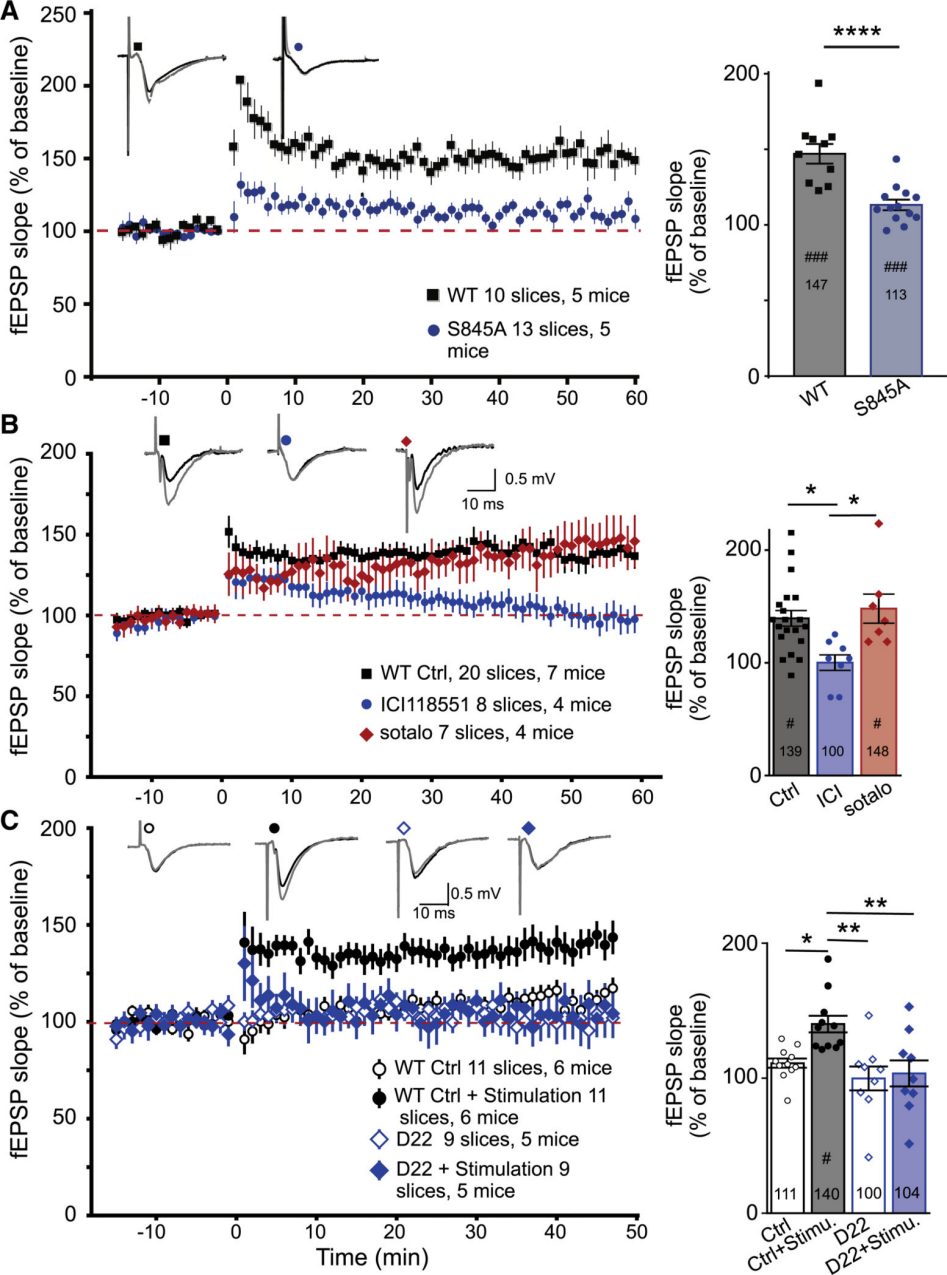
PKA-dependent HFS-LTP requires intracellular S845 phosphorylation Field EPSPs (fEPSPs) were recorded from acute hippocampal slices from wild-type (WT) and S845A KI mice before and after a single 100-Hz, 1-s tetanus at time point 0. Plots show the time courses of the initial slopes. Drug application, if indicated, started 10 min prior to tetanus and terminated with tetanus. Top left insets: sample traces of fEPSP recordings 5 min prior to tetanus (black lines) and 40 min post-tetanus (gray lines). Right insets: bar graphs of potentiation of fEPSPs 40–45 min after tetanus. Shown are means ± SEM. The exact values of the level of fEPSP after potentiation are given inside bars. Paired t test was used to determine significant differences of fEPSP slope averages from 5 to 0 min prior to tetanus and 40–45 min post-PTT (^#^*p* < 0.05 and ^###^*p* < 0.001). (A) HFS-LTP was strongly blunted in S845A KI versus WT mice (****p < 0.0001, t test). (B) The membrane-permeant β_2_AR-selective antagonist ICI118551 (100 nM) but not the membrane-impermeant, general βAR antagonist sotalol (100 μM) blocked induction of HFS-LTP (*p < 0.05 and **p < 0.01; one-way ANOVA followed by Dunnett’s multiple comparisons test). (C) D22 (100 nM) blocked induction of HFS-LTP but did not affect baseline control responses, which were recorded in parallel, alternating with potentiated pathway in a two-pathway configuration, over the full time period (*p < 0.05 and **p < 0.01; one-way ANOVA followed by Dunnett’s multiple comparisons test).

LTP induced by a single tetanus of 100 Hz that is 1 s long (or 2–3 tetani of 100 Hz that are ≥2 min apart) is blocked by pharmacological and genetic inhibition of PKA.^14,30,31,76,77^ This 1 × 100 Hz HFS-LTP does not require the addition of exogenous NE. We hypothesized that the baseline activity of PKA required for the 1 × 100 Hz HFS-LTP is driven by endogenous NE from norepinephrinergic terminals, which are preserved in acute hippocampal slices. Analogous to PTT-LTP, we thought that NE transport by OCT3 and PMAT into dendrites and the ensuing stimulation of the β_2_AR, and thereby S845 phosphorylation and GluA1 surface insertion, might be required for the 1 × 100 Hz HFS-LTP. In fact, we found that this HFS-LTP is nearly completely absent on slices from S845A knockin (KI) mice (Figure 5A). This finding indicates that HFS-LTP depends on S845 phosphorylation, like PTT-LTP except for HSF-LTP requiring endogenous NE, which supports basal PKA activity under non-stimulated conditions, is sufficient when for PTT-LTP a higher concentration of NE (i.e., addition of exogenous NE) is required.

**Figure 6.**
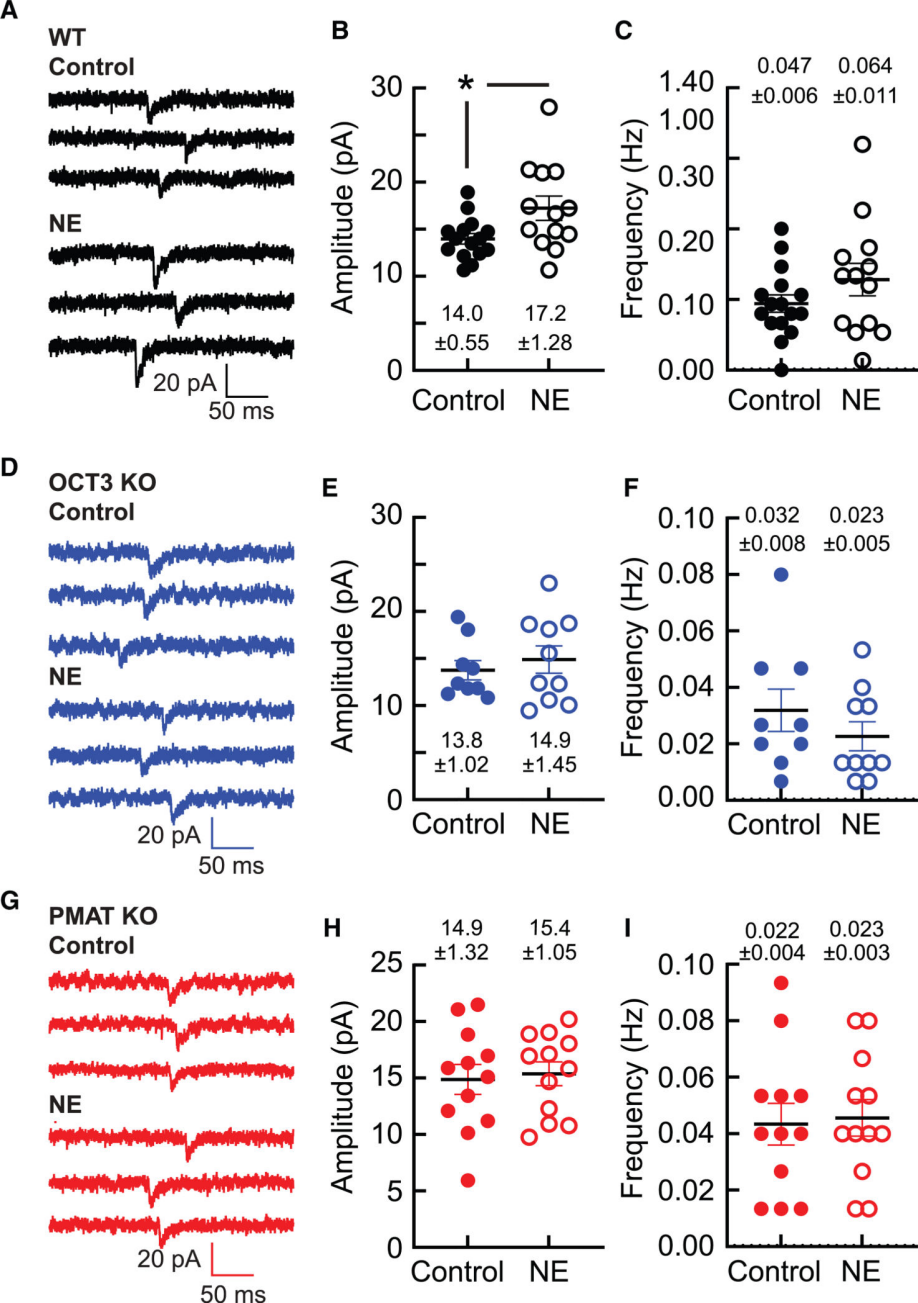
Injection of NE significantly augments mEPSC amplitude in WT, but not OCT3 and PMAT KO, mice Mini EPSCs (mEPSCs) were recorded from hippocampal CA1 pyramidal neurons in acute slices from 13- to 25-day-old WT (top, black traces), OCT3 KO (middle, blue traces), and PMAT KO mice (bottom, red traces). (A–C) Traces from representative mEPSC recordings without (top 3 traces) and with (bottom 3 traces) 20 μM NE in the recording electrode. (D–I) Dot plots of averages of mEPSC amplitudes (D–F) and frequencies (G–I). Dots represent means ± SEM of each individual experiment. Numbers underneath dots indicate overall means ± SEM of these averages. Only recordings from WT, but not OCT3 or PMAT KO, neurons show a clear increase in mEPSC amplitude that is statistically significant (*p < 0.05, Mann-Whitney U test).

Critically, the 1 × 100 Hz HFS-LTP was blocked by the membrane-permeant β_2_AR antagonist ICI118551 but not the membrane-*impermeant* β_2_AR antagonist sotalol (Figure 5B). These results indicate that this HFS-LTP, like PTT-LTP, requires signaling by β_2_ARs that are inside cells. We tested this hypothesis further by blocking OCT3 and PMAT with D22, which also blocked this HFS-LTP (Figure 6C). Accordingly, 1 × 100 Hz HFS-LTP also depends on NE transport into neurons and, thus, signaling by the β_2_ARs inside cells.

### Deletion of OCT3 and PMAT inhibits the increase in mEPSC amplitude by NE injection

To further evaluate whether NE can engage signaling by β_2_ARs that are inside neurons, NE was injected into CA1 pyramidal neurons during whole-cell patch recordings. In slices from 7- to 14-week-old mice, bath-applied NE increased fEPSP slopes by only 10% without reaching statistical significance (Figure S2A). Therefore, injection experiments were performed in slices from 2- to 3-week-old mice, an age at which β_2_AR stimulation consistently increased EPSC and miniature EPSC (mEPSC) amplitudes in our earlier work.^9^ Inclusion of NE in the patch pipet induced a statistically significant increase in mEPSC amplitude by 22% over time, likely reflecting the time needed for NE diffusion throughout the dendrites (Figures 6A–6C). These results suggest that NE can induce signaling via the β_2_ARs inside cells. However, the NE binding site of the β_2_ARs in intracellular vesicles is on the luminal side of the β_2_AR, requiring NE not only to be present in the cytosol but also to be transported across the vesicular membrane. OCT3 and PMAT are not coupled to any co-transport or ATP activity and can readily transport substrates in both directions. To test whether OCT3, PMAT, or both are required for efficient transport of NE into vesicles, we repeated NE injection in OCT3 and PMAT KO slices. The increase in mEPSC amplitude during perfusion with NE was minimal in each of the transporter KO genotypes (8% for OCT3 KO and 3% for PMAT KO neurons; Figures 6D–6I). Neither of these two increases reached statistical significance, which clearly contrasts with the statistically significant increase in wild-type (WT) mice. These data suggest that in neurons, both transporters contribute to the uptake of NE into the vesicles.

To ensure that sotalol did effectively block signaling at the cell surface, we tested whether sotalol reduced GluA1 S845 phosphorylation when NE is included in the bath solution for optimal stimulation of surface β_2_ARs. The S845 phosphorylation seen with NE alone was significantly decreased when sotalol was applied together with NE (Figures S4D and S4E).

The above findings with sotalol show that signaling by β_2_ARs inside cells can be directly measured by systematically blocking the signaling of β_2_ARs on the cell surface with sotalol. Thus, to further evaluate the role of OCT3 and PMAT in regulating S845 phosphorylation of GluA1 in the cell interior, we incubated slices from WT, OCT3 KO, and PMAT KO mice with NE versus vehicle with sotalol present under both conditions. NE significantly stimulated S845 phosphorylation in forebrain slices from WT mice (Figures S4F and S4G; *p* = 0.027) but not from OCT3 KO or PMAT KO mice (Figures S4F and S4G; the trend of NE augmenting S845 phosphorylation in the KO slices was, in both genotypes, far from reaching significance: *p* = 0.287 for PMAT KO and *p* = 0.264 for OCT3 KO).

We conclude that NE can upregulate postsynaptic AMPAR responses by acting inside dendrites upon its transport by OCT3 and PMAT first into the cytosol and then into the lumen of the secretory vesicles.

## DISCUSSION

For NE to mobilize intracellular AMPARs when cAMP signaling is restricted to individual β_2_AR-AMPAR complexes,^9,47,48^ NE has to enter neurons and, ultimately, the lumen of the AMPAR storage vesicles, where the ligand binding sites of intracellular β_2_ARs reside. Inspired by recent findings suggestive of NE and dopamine uptake by system 2 cation transporters and of intracellular signaling in HEK293 cells,^52,53,56,57^ we analyzed the effects of inhibitors of OCT3 and PMAT and their deletion on AMPAR phosphorylation, surface localization, and LTP. The results advance our mechanistic understanding of neuronal functions by elucidating several important aspects of NE signaling.

Firstly, the partial block of NE-induced phosphorylation of GluA1 S845 by three different OCT3 and PMAT inhibitors indicates that NE stimulates S845 phosphorylation not only on the surface but also inside neurons after it has been transported into the cell.

Secondly, NE-induced surface insertion of AMPARs from an intracellular reservoir is fully blocked by OCT3 and PMAT inhibitors and, thus, depends on active OCT3 and PMAT transport of NE. This finding is consistent with the model that regulation of GluA1 S845 phosphorylation is mediated in a highly localized manner by β_2_ARs that are associated with AMPARs in an intracellular compartment,^9^ which then promotes secretory trafficking of these AMPARs to the cell surface through yet-to-be-defined molecular mechanisms.

Thirdly, PTT-LTP induced via membrane-impermeant NE is blocked by all three pharmacological inhibitors of OCT3 and PMAT and is largely abrogated in OCT3 as well as PMAT KO mice. That PTT-LTP induced with membrane-permeant ISO is not at all affected under any of these conditions indicates that the effects on NE-induced PTT-LTP are due to the loss of NE transport and not of other molecular mechanisms that are important for the plasticity changes. Similarly, HFS-LTP induced by a single 1-s, 100-Hz tetanus was blocked by the highly potent OCT3/PMAT inhibitor D22 and also the membrane-permeant β_2_AR antagonist ICI118551 but not the membrane-impermeant β_2_AR antagonist sotalol. Collectively, these findings define a hitherto unrecognized function of NE transport into neurons by system 2 in synaptic plasticity in general and specifically in PKA-dependent forms of LTP.

Fourthly, we find that the 1 × 100 Hz HFS-LTP depends on S845, which defines a clear functional role of S845 phosphorylation in PKA-dependent forms of synaptic plasticity as suggested earlier based on ectopic overexpression of S845A mutant GluA1.^21^ However, S845 phosphorylation is not required for all forms of LTP (e.g., Granger et al.^78^). It might, thus, mediate a subset of cognitive functions rather than being a dominant determinant of many functions. Functions that might involve S845 phosphorylation include those that are governed specifically by strong NE signaling, such as learning in novel and emotionally charged situations.^1,5–8^ Consistently, a form of fear conditioning that depends on the pre-exposure of the mice to the shock context is deficient in S831A/S845A double KI (DKI) mice.^7^

OCT3 and PMAT are uniporters and transport NE down its concentration gradient, independent of other ion gradients. Importantly, they can operate in both directions.^54,69^ Operating in one direction, transporters that reside in the plasma membrane can transport NE from the cell exterior into the cytosol, and then, operating in their reverse direction, transporters that reside in the membrane of intracellular vesicles transport NE from the cytosol into the vesicle lumen (Figure S1A). OCT3 and PMAT might act in parallel or in series in the plasma membrane and AMPAR storage vesicles, such as REs.^33^ Thus, it is possible that one transports NE through the plasma membrane and the other one into REs. However, the NE-dependent PTT-LTP in adult mice and NE-induced upregulation of mEPSC amplitude in juvenile mice by NE do not appear to be completely abrogated in either OCT3 or PMAT KO mice. These results suggest that each transporter can, to some degree, mediate transport across both the plasma membrane and the vesicle membrane in this functional context. Alternatively, OCT2, which is also present in some neurons and whose elimination also has central effects,^57,61,69^ could contribute to some aspect of NE transport as well.

In general terms, the effects of blocking NE transport on PTT-LTP and HFS-LTP and the impairment in upregulation of mEPSC amplitude upon direct injection of NE into neurons in OCT3 and PMAT KO mice identify an important function of OCT3 and PMAT as regulators of both postsynaptic AMPAR localization and LTP, which in turn can explain central effects observed in the respective KO mice.^60,62^ The 5-Hz stimulus train used to induce PTT-LTP mimics theta oscillations, a prominent rhythmic activity of 4–8 Hz of hippocampal neurons, which is thought to be relevant for memory formation.^79,80^ That OCT3 and PMAT transport NE with low affinity but high capacity qualifies them as conduits for NE signaling, especially when extracellular NE levels are high. Thus, it is tempting to speculate that the requirement of OCT3 and PMAT for PTT-LTP reflects the role of these two transporters in memory formation of novel and emotional content, which, again, is driven by strong NE signaling.^1,5–8^ Such a function of OCT3 and PMAT would parallel the role of S845 phosphorylation proposed above, which would be consistent with these two processes being part of the same signaling pathways.

Notably, our HFS-LTP does not require the addition of exogenous NE to the hippocampal slices. Accordingly, endogenous NE that is released under basal conditions without specific stimulation can foster the induction of LTP under certain conditions. Because NE oscillates with circadian rhythmicity, it is conceivable that NE uptake by OCT3 and PMAT and the ensuing S845 phosphorylation are mediators of circadian variations in learning. In fact, circadian changes in S845 phosphorylation^81,82^ mirror the changes in NE content being high during active phases (which predominate at night for nocturnal animals like rats and mice) and low during inactive phases (which predominate during the day for nocturnal animals).^83–89^

### Limitations of the study

Several G-protein-coupled receptors (GPCRs) can undergo endocytosis with their endogenous (or pharmacological) ligands bound to them and then continue to signal inside cells from endocytic compartments. For instance, the thyroid-stimulating hormone receptor, the parathyroid hormone receptor, and the vasopressin receptor undergo endocytosis together with their cognate agonists to continue to stimulate cAMP production from intracellular sites.^49–51^ Other work indicates the co-endocytosis of GPCRs with fluorescent ligands.^90^ Furthermore, blocking endocytosis can prevent intracellular signaling by the β_2_ARs.^52,53,91,92^ Accordingly, it could be possible that NE-associated β_2_ARs reach AMPARs at intracellular compartments such as REs, which harbor AMPARs for surface trafficking,^33,93^ and stimulate their phosphorylation on S845. To obtain some provisional insight into this possibility, we infected hippocampal cultures from β_1_AR/β_2_AR double KO (DKO) mice with lentivirus to express either the WT β_2_AR or a β_2_AR mutant of PKA phosphorylation sites (S261A/S262A) or GPCR kinase (GRK) phosphorylation sites S355A/S356A; the latter (but not the former) renders it endocytosis deficient,^94^ similar to the “β_2_-AR-3S” mutant in Irannejad et al.^52^ ISO induced S845 phosphorylation to the same degree in the GRK mutant as in the WT and also the PKA mutant β_2_ARs (Figure S5A). Furthermore, we utilized forebrain slices from the S355A/S356A DKI mice to test the effect of NE. Like ISO, NE stimulated S845 phosphorylation to the same degree in DKI slices as in WT slices (Figure S5B). These findings contrast with the statistically significant reductions in NE-induced S845 phosphorylation by the three different OCT3 and PMAT blockers (Figure 1). Accordingly, co-endocytosis of NE with the β_2_ARs does not play an immediately prominent role in intracellular S845 phosphorylation, although a subtler role cannot be excluded. Of note, inhibition of intracellular β_2_AR signaling upon blocking endocytosis^52,53,91,92^ can also be explained by depletion of the β_2_ARs from the relevant intracellular pools, like REs.

It would be desirable to define the exact localization of OCT3 and PMAT inside dendrites. In a first attempt, we obtained all commercially available antibodies: four antibodies against OCT3 and two antibodies against PMAT. Although some antibodies showed immunofluorescence staining signals in dendrites of WT neurons, comparable staining was still present in respective KO neurons, suggesting that the staining in WT neurons is largely non-specific (Figure S6). Ectopic expression of EGFP-tagged OCT3 and PMAT as an alternative approach was not tolerated in our hippocampal cultures. However, we were able to express EGFP-OCT3 and EGFP-PMAT in HEK293 cells, a well-established model system for the analysis of cell biology and signaling by βARs. Both transporters were clearly detectable inside cells even after a 4-h treatment with cycloheximide to inhibit protein translation and to chase any newly synthesized EGFP-OCT3 or EGFP-PMAT protein out of the early secretory pathway, i.e., the endoplasmic reticulum and Golgi apparatus. This intracellular localization was very prominent and dominant for PMAT, as seen in individual mid-level z sections (Figure S7A). There was substantial overlap between PMAT and the immunofluorescent signals for endogenous Rab11a, which labels one of presumably several populations of REs. Remarkably, OCT3 was much more pronounced at and near the cell periphery than in the cell interior (Figure S7B), in striking contrast to PMAT. However, there were clear examples where OCT3 colocalized with Rab11a in the interior, albeit close to the plasma membrane. That the overlap of PMAT and OCT3 was far from complete suggests that OCT3 and PMAT are present in compartments that are not labeled by Rab11a and, at the same time, are by far not in all Rab11-positive compartments. Thus, beyond this initial impression, the determination of the exact subcellular localization of OCT3 and PMAT in dendrites has to wait until specific antibodies become available.

### RESOURCE AVAILABILITY

#### Lead contact

Requests for further information, resources, and reagents should be directed to the lead contact, Johannes W. Hell (jwhell@ucdavis.edu).

#### Materials availability

This study did not generate new reagents.

#### Data and code availability

- All data in this study are available either within the article or from the lead contact upon request.
- This study did not generate new code.
- Any additional information required to reanalyze the data reported in this paper is available from the lead contact upon request.

## STAR★METHODS

### EXPERIMENTAL MODEL AND STUDY PARTICIPANT DETAILS

#### Animals

All animal procedures strictly followed NIH guidelines and were approved by the UC Davis Institutional Animal Care and Use Committee (IACUC; Protocol #18837 and #22403). The *Animal Care Unit* at UC Davis is responsible for the housing and care of all animals including rats and mice in a highly control environment that is optimized for the comfort of rodents. This facility is AAALAC accredited (the NIH Office of Laboratory Animal Welfare (OLAW) Assurance Number is A3433–01).

##### Mice

WT and all KO mice were on a strict C57BL/6J genetic background. The KO mice were exactly as described for β_1_ AR/β_2_ AR DKO,^94^ OCT3,^96^ and PMAT.^97^ Gene pools were systematically refreshed every 3 years with outside WT C57BL/6J mice from Jackson Labs. All mice were of both sexes and 7–20 weeks of age except for mEPSC recordings when slices were prepared from P13-P25 mice, and for hippocampal cultures, which were prepared from P1 mice of both sexes.

##### Rats

E18 embryonic Sprague-Dawley rats of both sexes were used to prepare primary hippocampal cultures (from timed pregnant female directly obtained from Charles River Laboratories).

##### Limitation

It was not possible to determine effects of sex because the complexity of the experiments required a large number of repetitions to obtain enough power to determine just drug and genotype effects.

#### Cell cultures

##### Hippocampal cultures

Hippocampal cultures (HC) were prepared from either P0-P1 newborn WT, β_1_ AR/β_2_ AR DKO, OCT3 KO, and PMAT KO mice or E18 embryonic Sprague-Dawley rats of both sexes.

##### HEK 293 cells

HEK293T/17 cells were verified by and directly obtained from ATCC. Multiple frozen aliquots of early passage cultures (P2 or P3) were immediately prepared for storage in liquid nitrogen as original source for our cell cultures. The health of the HEK293 cell cultures was routinely determined by trypan blue stain discernment of live/dead cell counts to ensure >95% viability.

### METHOD DETAILS

#### Reagents, peptides, and antibodies

NE bitartrate salt and ISO bitartrate salt were from Sigma, EGTA, EDTA, Tween 20, Triton X-100, and tris(hydroxymethyl)aminomethane (Tris) from Thermo Fisher Scientific, and ECL reagents from Millipore and Thermo Fisher Scientific. Microcystin LR was from Calbiochem, protein A-coated beads from Repligen, protein G-coated beads from GE Healthcare, and polyvinyldene fluoride (PVDF) membranes from Millipore. Antibodies against GluA1 C terminus, phosphoS831, phosphoS845 are as in Patriarchi et al. and Zhang et al.^46,95^ The antibody against the GluA1 N terminus was from Calbiochem (PC246), PSD-95 from NeuroMab (clone K28/43) and MAP2B from BD Transduction Laboratories. Other reagents were obtained from established suppliers and of standard quality.

#### Preparation of brain slices

7–20 week old mice were decapitated and brains placed into ice-cold slicing artificial cerebrospinal fluid (slicing ACSF; in mM: 10 NaCl, 230 sucrose, 26 NaHCO_2_, 1.2 KH_2_PO_4_, 2.5 KCl, 1 CaCl_2_, 1.3 MgSO_4_ and 10 D-glucose, 290–300mOsm/kg, saturated with 95% O_2_ and 5% CO_2_; final pH 7.3). ∼1/3 of the rostral and caudal portions of the brain were trimmed off. 400 μm thick forebrain slices containing hippocampus were prepared with a vibratome (Leica VT 1000A) and equilibrated in ACSF (ACSF; in mM: 126 NaCl, 26 NaHCO_2_, 1.2 KH_2_PO_4_, 2.5 KCl, 1 CaCl_2_, 1.3 MgSO_4_ and 10 D-glucose, 290–300mOsm/kg, saturated with 95% O_2_ and 5% CO_2_; final pH 7.3) for 1 h at 32°C before use.

#### Biochemistry

Slices and hippocampal cultures (see next paragraph) were treated with the indicated drugs for 5 min and homogenized by trituration with insulin syringes in a 10-fold excess (volume/weight) of immunoprecipitation (IP) buffer (1% Triton X-100, 150 mM NaCl, 10 mM EDTA, 5 mM EGTA, 50 mM Tris-HCl, pH 7.4)^98^ containing protease inhibitors (10 μg/mL pepstatin A, 1 μg/mL leupeptin, 2 μg/mL aprotinin and 200 nM PMSF, the latter being added immediately before homogenization and again at the start of IP), and phosphatase inhibitors (4 μM microcystin LR, 1 mM *p*-nitrophenyl phosphate, 25 mM Na-pyrophosphate, and 25 mM NaF).^99,100^ Non-solubilized material was removed by ultracentrifugation (250,000 × g for 30 min) before IP (4 h at 4°C) with antiserum against GluA1 C terminus (1 μL) or Rabbit control IgG (Thermo Fisher Scientific) using equal amounts of protein A and G Sepharose. After three washes with TBS samples underwent SDS-PAGE and transfer onto PVDF membranes. Immunoblots (IBs) were blocked with 3% BSA in TBS plus 0.1% Tween-20 (TBST), incubated with primary antibodies (3% BSA in TBST; 3 h), washed three times with TBST, incubated with HRP-conjugated secondary antibody (1:10,000 in 3% BSA in TBST; 1 h), and washed several times for 30 min before detection of HRP signals with ECL or ECL plus chemiluminescence reagents by film exposure. Multiple exposures of increasing length ensured that signals were in the linear range, as described.^99,101^

#### Hippocampal cultures

Dissociated hippocampal cultures (HC) were prepared from early postnatal day (P0-P1) mice or embryonic E18 rats as described.^102^ Briefly, after careful but swift trituration of hippocampi, neurons were plated on coverslips (25 mm, #1.5) coated with poly-L-lysine at a density 200,000/well in 6-well plates. Cultures were maintained in Neurobasal medium, supplemented with 1× N21-Max, 1× GlutaMax™ Supplement, 5% heat inactivated fetal bovine serum (FBS), and 1 μg/mL gentamycin in a humidified 37°C incubator with 5% CO_2_ until 20–22 DIV.

#### HEK 293 cells

Passage 2 HEK293T cells were cultured from frozen stock and maintained in high glucose Dulbecco’s modified Eagle’s medium (GIBCO -ThermoFisher Scientific) supplemented with 10% FBS, 2mM L-Glutamine and 100 μg/mL penicillin + streptomycin (Pen/Strep) at 37°C in 5% CO_2_ and 95% air. The health of the HEK293 cell cultures was routinely determined by trypan blue stain discernment of live/dead cell counts to ensure >95% viability. Less than 95% viability cultures were discarded and replacement passage 2 aliquots thawed, cultured, expanded and maintained for routine use. Cell cultures were split and passaged ∼15 to maximum 20 times before starting a new early passage culture. HEK293T/17 cells were transfected with pcDNA3 encoding either EGFP or C-terminally EGFP-tagged OCT3 or PMAT using JetPrime reagent and fixed 24 h post transfection.

#### Immunocytochemistry

DIV 20–22 HCs and HEK293 cells were fixed with PBS containing 4% paraformaldehyde (PFA) and 4% (w/v) sucrose at room temperature (RT) for 5 min followed by blocking with blocking solution (PBS containing 50 mM NH_4_Cl, 2% glycerol, 2% normal goat serum (Jackson ImmunoResearch), 5% fetal bovine serum (Atlanta Biologicals)) at RT for 2 h. The neurons were stained with the antibody against the GluA1 N terminus at RT for 2 h, washed three times with PBS, and permeabilized in PBS containing 0.25% Trition X-100 at RT for 8 min for intracellular protein staining. After washing coverslips twice with PBS containing 0.01% Trition X-100 (PBST) and incubation with blocking solution at RT for 2 h, antibodies against PSD-95 and MAP2B were applied to neurons for 2 h at RT or overnight at 4°C. Coverslips were washed with PBST three times, incubated as before with blocking solution, and cells were labeled with fluorophore-conjugated secondary antibodies for 2 h at RT. Staining with antibodies against OCT3, PMAT and Rab11a was performed in the same way after permeabilization with 0.25% Trition X-100. Fluorescence images were acquired by a Leica SP2 confocal microscope after washing with PBST, PBS, and MilliQ water and sealing with antifade mountant (PermaFluor, ThermoScientific). The confocal images were analyzed by ImageJ software.

#### Quantification and statistical analysis of biochemical and immunocytochemical data

Data are expressed as means ± standard error of the mean (SEM) and analyzed with Excel (Microsoft Corp.) and GraphPad Prism software (GraphPad, San Diego, CA). For normally distributed data, the one-way ANOVA test was used to analyze the statistical difference between more than two different conditions followed by Tukey’s or uncorrected Fisher’s LSD post-hoc test. We performed planed comparisons of predetermined sample pairs for the relevant pairs, i.e., NE versus control, NE versus inhibitors, and control versus inhibitors alone. For nonparametric data, the Kruskal-Wallis test and Dunn’s multiple comparisons test were used. When the *p* value is lower than 0.05, data are considered significantly different and the significance is represented by asterisks (*) in the figures (*p < 0.05, **p < 0.01, ***p < 0.001).

#### Field EPSP recording from brain slices

Recordings were as described previously.^32,46^ Briefly, 400 μm thick coronal forebrain slices were prepared from 7–14 week old WT, OCT3 KO, or PMAT KO mice in the C57BL6/J genetic background. The buffer used during slice preparation was (in mM) 2.5 KCl, 3 MgSO_4_, 1.2 NaH_2_PO_4_, 26 NaHCO_3_, 10 dextrose, 220 sucrose, 1.1 CaCl_2_. Following slicing, the individual samples were incubated for at least one 1 h at 32°C before transfer to the recording chamber. Slices were perfused with a flow rate of 2 mL/min at 33°C with field recording ACSF (frACSF; in mM: 127 NaCl, 1.9 KCl, 1 MgSO_4_, 1.2 NaH_2_PO_4_, 26 NaHCO_3_, 10 dextrose, 2.2 CaCl_2_) equilibrated with 95% O_2_ and 5% CO_2_ (final pH 7.3) using a peristaltic pump for recirculation. Schaffer collaterals were stimulated every 15 s with a tungsten concentric bipolar microelectrode and the resulting fEPSPs in the CA1 region were recorded using a glass electrode filled with frACSF. The stimulation electrode was placed in stratum radiatum near the recording electrode placed in stratum pyramidale in CA1. For simultaneous two-pathway recordings, two stimulation electrodes were placed on both sides of the recording electrode and stimulation was alternated every 10 s. Signals were amplified (Axopatch 1D, Axon Instruments), digitized (Digidata 1320A, Axon Instruments), and analyzed (Clampex 9, Molecular Devices). Stimulus strength was titrated (0.2–1.0 mV) for each slice and the resulting responses were used to determine IOR as a function of the stimulus strength to the maximal response of the resulting fEPSP. Subsequent stimulus strength was ∼50% of the maximal response seen in the IOR for the rest of the experiment. Paired pulse facilitation (PPF) was determined as the initial slopes of fEPSPs responses following two consecutive, single-pulse stimuli with interstimulus intervals of 10, 20, 50, 100, 200, and 500 ms. To determine IORs for AMPAR and NMDAR responses, fiber volley amplitudes and initial fEPSP slopes were measured in frACSF (AMPAR) or frACSF with no Mg^2+^ but 10 μM NBQX with increasing stimulus strengths (NMDAR). For LTP, baseline recordings were produced by recording fEPSPs every 15 s for ∼15 min before any drug treatment, which started 5 min prior to stimulation at 5 Hz for 3 min (PTT-LTP) or 100 Hz for 1s (1 × 100Hz/1s HFS-LTP). Drug application was stopped at the end of the tetani. fEPSPs were recorded for 50–60 min after the end of the tetani. To define the magnitude of LTP, the average of fEPSP initial slopes from the 5 min preceding the tetanus was determined and set to correspond to 100% baseline level. The average of fEPSP initial slopes obtained between 45 and 50 min after the tetanus was calculated to determined potentiation relative to the 100% baseline.

#### Whole-cell patch-clamp recording of mEPSCs

Slices were prepared from P13–25 old mice as described for fEPSP recordings and perfused at room temperature with oxygenated patch recording ACSF (prACSF; in mM: 119 Nacl, 3 KCl, 1.3 MgSO_4_, 1.25 NaH_2_PO_4_, 26 NaHCO_3_, 2.5 CaCl_2_, 11 glucose, 0.001 tetrodotoxin, 0.03 bicuculline). Pyramidal neurons in the CA1 region were visually identified using an Olympus BX50WI upright microscope and an Olympus LumPlanFL 40× water-immersion objective with IR-DIC contrast through a Hamamatsu C2400 CCD camera. Patch micropipettes (2.5–5 MΩ) were filled with intracellular solution (in mM: 115 CsMeSO_4_, 10 CsCl, 5 NaCl, 20 HEPES, 5 Mg-ATP, 0.3 Na-GTP, 4 ascorbic acid, 10 EGTA; pH 7.2) containing either vehicle (H_2_O) or 20 μM NE. Patch-clamp recordings were made in whole-cell configuration using Clampex 9 to control an MultiClamp 700B amplifier (Axon Instruments) through a Digidata 1322A digitizer. Cell capacitance and series resistance were observed but not compensated throughout experiments. The holding potential was −70 mV and signals were sampled at 10 kHz using a 1 kHz low-pass filter. Events were identified using template-based event detection function of Stimfit and confirmed by manual observation. Per-cell averages were used for statistical comparison between genotypes using Mann-Whitney U test. Analysis was performed using Microsoft Excel and GraphPad Prism.

## Supplementary Material

1

SUPPLEMENTAL INFORMATION

Supplemental information can be found online at https://doi.org/10.1016/j.celrep.2025.116011.

## Figures and Tables

**Table:** KEY RESOURCES TABLE

REAGENT or RESOURCE	SOURCE	IDENTIFIER
Antibodies		
Rabbit polyclonal anti-GluA1 C terminus	Patriarchi et al.^46^; Zhang et al.^95^	Produced by JWH
Rabbit polyclonal anti-phosphoS831	Patriarchi et al.^46^; Zhang et al.^95^	Produced by JWH
Rabbit polyclonal anti-phosphoS845	Patriarchi et al.^46^; Zhang et al.^95^	Produced by JWH
Rabbit polyclonal anti- GluA1 N terminus (PC246)	Calbiochem	Discontinued. Replaced by Millipore – ABN241
Mouse monoclonal anti-PSD95 (clone K28/43)	NeuroMab	https://www.antibodyregistry.org/AB_2877189
Mouse monoclonal anti-MAP2B	BD Transduction Laboratories	https://www.antibodyregistry.org/AB_397833
Mouse monoclonal anti-EEA1	BD Transduction Laboratories	https://www.antibodyregistry.org/AB_397830
Rabbit monoclonal anti-Rab11a	Invitrogen	https://www.antibodyregistry.org/AB_3074855
Rabbit monoclonal anti-OCT3	Alpha Diagnostics Intl	Cat#OCT31-A
Rabbit polyclonal anti-OCT3	Novus	Cat# NBP1–83976-25
Rabbit monoclonal anti-OCT3	Novus	Cat# NBP3–22312-50
Rabbit monoclonal anti-OCT3	Biossusa	Cat# BSM-54709R
Rabbit monoclonal anti-PMAT	Alomone Labs	Cat#ANT-054; https://www.antibodyregistry.org/AB_2925114
Rabbit monoclonal anti-PMAT	Sigma Aldrich	Cat# SAB1300519–100ug; https://www.antibodyregistry.org/AB_10606061
Peroxidase IgG Fraction Monoclonal Mouse Anti-Rabbit IgG, Light Chain Specific	Jackson ImmunoResearch	211–032-171
Alexa 488- anti-ms IgG2a	Thermo Fisher Scientific	A-21131
Alexa 555 - anti-Rb IgG1	Thermo Fisher Scientific	A32732
Alexa 647- anti-ms IgG1	Thermo Fisher Scientific	A55060
Alexa 647 - anti-Rb IgG2B	Thermo Fisher Scientific	A-21242
Chemicals, peptides, and recombinant proteins		
(±)-Norepinephrine (+)-bitartrate salt	Sigma Aldrich	A0937
(−)-Isoproterenol hydrochloride	Sigma Aldrich	I6504
Decynium 22	TOCRIS	4722
Corticosterone	Sigma Aldrich	C2505
Lopinavir, 98%, Thermo Scientific Chemicals	Thermo Fisher Scientific	AC456750010
Desipramine hydrochloride	TOCRIS	3067
Sotalol hydrochloride	TOCRIS	0952
EGTA	Research Product International	E57060–50.0
EDTA	Sigma-Aldrich	SLBS2513V
Tween-20	Sigma-Aldrich	P2287–100ML
Triton X-100	Sigma-Aldrich	X100–500ml
Tris Base Ultra Pure [Tris (Hydroxymethyl) Aminomethane]	Research Product International	RPI-T60040–25000.0
Immobilon Classico Western HRP substrate	Millipore Sigma	WBLUC0500
Immobilon Crescendo Western HRP substrate	Millipore Sigma	WBLUR0500
SuperSignal™ West Femto Maximum Sensitivity Substrate	Fisher Scientific	34096
Microcystin LR	Calbiochem	475815 (Discontinued)
Pepstatin A	Cayman Chemical	9000469
leupeptin	Fisher Scientific	78435
aprotinin	Sigma-Aldrich	A1153–10MG
PMSF	Millipore Sigma	10837091001
p-nitrophenyl phosphate	Sigma-Aldrich	4876–1GM
Na-pyrophosphate	Sigma-Aldrich	P8010–500G
NaF	Sigma-Aldrich	201154–100G
CaptivA^®^ Protein A Affinity Resin	Repligen	CA-PRI-1000
Protein G Sepharose 4 Fast Flow	Millipore Sigma	GE17–0618-01
polyvinyldene fluoride (PVDF) membranes	BIO-RAD	1620177
NaCl	Thomas Scientific	C987L20 (EA/1)
Sucrose	Thomas Scientific	C791V78 (EA/1)
NaHCO_3_	Fisher Scientific	S233–3
KH_2_PO_4_	Fisher Scientific	P285–3
KCl	Fisher Scientific	AA11595A1
CaCl_2_	Fisher Scientific	AAL131910B
MgSO_4_	Spectrum Chemical	M1063
D-glucose	Fisher Scientific	AAA1682836
Triton X-100	Fisher Scientific	AAA16046AP
BSA	Fisher Scientific	23209
paraformaldehyde	Thomas Scientific	C993M32 (EA/1)
NH4Cl	Fisher Scientific	A2037300G
glycerol	Fisher Scientific	BP229–1
normal goat serum	Fisher Scientific	16210072
fetal bovine serum (FBS)	Atlanta Biologicals (now R&D Systems)	S11150
Prolong Diamond Mountant	Fisher Scientific	P36965
Neurobasal medium	Fisher Scientific	21103049
1x GlutaMax™ Supplement	Fisher Scientific	35050061
PermaFluor Mountant	Fisher Scientific	P10144
1x N21-Max	R&D Systems	AR008
gentamycin	Sigma-Aldrich	G1272
poly-L-lysine	Peptide Institute	3056
DMEM Medium	Gibco/Fisher Scientific	11965092
jetPrime Polyplus Transfection Reagent	Sartorius	101000046
Experimental models: Cell lines		
HEK293T/17	ATCC	CRL-3216
Experimental models: Organisms/strains		
Mouse: Oct3 KO	Zwart et al.^96^	N/A
Mouse: Pmat KO	Duan and Wang^97^	N/A
Mouse: beta 1/beta 2 adrenergic receptor double KO	Shen et al.^94^	N/A
Recombinant DNA		
Plasmid: SLC29A4_pcDNA3.1(+)-C EGFP	Genewiz	Custom Production
Plasmid: SLC22A3_pcDNA3.1(+)-C EGFP	Genewiz	Custom Production
Other		
Coverslips	Warner Instruments	64–0715

## Highlights

- Norepinephrine (NE) stimulates β_2_ARs that reside inside neurons
- NE is transported into neurons and lumen of secretory vesicles by OCT3 and PMAT
- The resulting phosphorylation of AMPARs by PKA stimulates their surface insertion
- This intracellular signaling by NE is required for PKA-dependent forms of LTP

## References

[1] Cahill L, Prins B, Weber M, and McGaugh JL (1994). Beta-adrenergic activation and memory for emotional events. Nature 371, 702–704. 7935815 10.1038/371702a0

[2] Strange BA, and Dolan RJ (2004). Beta-adrenergic modulation of emotional memory-evoked human amygdala and hippocampal responses. Proc. Natl. Acad. Sci. USA 101, 11454–11458. 15269349 10.1073/pnas.0404282101 PMC509222

[3] Strange BA, Hurlemann R, and Dolan RJ (2003). An emotion-induced retrograde amnesia in humans is amygdala- and beta-adrenergic-dependent. Proc. Natl. Acad. Sci. USA 100, 13626–13631. 14595032 10.1073/pnas.1635116100 PMC263864

[4] Nielson KA, and Jensen RA (1994). Beta-adrenergic receptor antagonist antihypertensive medications impair arousal-induced modulation of working memory in elderly humans. Behav. Neural. Biol 62, 190–200. 7857241 10.1016/s0163-1047(05)80017-2

[5] Berman DE, and Dudai Y (2001). Memory extinction, learning anew, and learning the new: dissociations in the molecular machinery of learning in cortex. Science 291, 2417–2419. 11264539 10.1126/science.1058165

[6] Minzenberg MJ, Watrous AJ, Yoon JH, Ursu S, and Carter CS (2008). Modafinil shifts human locus coeruleus to low-tonic, high-phasic activity during functional MRI. Science 322, 1700–1702. 19074351 10.1126/science.1164908

[7] Hu H, Real E, Takamiya K, Kang MG, Ledoux J, Huganir RL, and Malinow R (2007). Emotion Enhances Learning via Norepinephrine Regulation of AMPA-Receptor Trafficking. Cell 131, 160–173. 17923095 10.1016/j.cell.2007.09.017

[8] Carter ME, Yizhar O, Chikahisa S, Nguyen H, Adamantidis A, Nishino S, Deisseroth K, and de Lecea L (2010). Tuning arousal with optogenetic modulation of locus coeruleus neurons. Nat. Neurosci 13, 1526–1533. 21037585 10.1038/nn.2682 PMC3174240

[9] Joiner M.l.A., Lisé MF, Yuen EY, Kam AYF, Zhang M, Hall DD, Malik ZA, Qian H, Chen Y, Ulrich JD, (2010). Assembly of a beta2-adrenergic receptor-GluR1 signalling complex for localized cAMP signalling. EMBO J. 29, 482–495. 19942860 10.1038/emboj.2009.344 PMC2824466

[10] Wang D, Govindaiah G, Liu R, De Arcangelis V, Cox CL, and Xiang YK (2010). Binding of amyloid beta peptide to beta2 adrenergic receptor induces PKA-dependent AMPA receptor hyperactivity. FASEB J. 24, 3511–3521. 20395454 10.1096/fj.10-156661 PMC2923357

[11] Zhang M, Patriarchi T, Stein IS, Qian H, Matt L, Nguyen M, Xiang YK, and Hell JW (2013). Adenylyl Cyclase Anchoring by a Kinase Anchor Protein AKAP5 (AKAP79/150) Is Important for Postsynaptic β-Adrenergic Signaling. J. Biol. Chem 288, 17918–17931. 23649627 10.1074/jbc.M112.449462 PMC3682589

[12] Purkey AM, Woolfrey KM, Crosby KC, Stich DG, Chick WS, Aoto J, and Dell’Acqua ML (2018). AKAP150 Palmitoylation Regulates Synaptic Incorporation of Ca^2+^-Permeable AMPA Receptors to Control LTP. Cell Rep. 25, 974–987.e4. 30355502 10.1016/j.celrep.2018.09.085 PMC6263960

[13] Purkey AM, and Dell’Acqua ML (2020). Phosphorylation-Dependent Regulation of Ca^2+^-Permeable AMPA Receptors During Hippocampal Synaptic Plasticity. Front. Synaptic Neurosci 12, 8. 32292336 10.3389/fnsyn.2020.00008 PMC7119613

[14] Sanderson JL, Gorski JA, and Dell’Acqua ML (2016). NMDA Receptor-Dependent LTD Requires Transient Synaptic Incorporation of Ca^2+^-Permeable AMPARs Mediated by AKAP150-Anchored PKA and Calcineurin. Neuron 89, 1000–1015. 26938443 10.1016/j.neuron.2016.01.043 PMC4914360

[15] Sanderson JL, Scott JD, and Dell’Acqua ML (2018). Control of Homeostatic Synaptic Plasticity by AKAP-Anchored Kinase and Phosphatase Regulation of Ca^2+^-Permeable AMPA Receptors. J. Neurosci 38, 2863–2876. 29440558 10.1523/JNEUROSCI.2362-17.2018 PMC5852664

[16] Tavalin SJ, Colledge M, Hell JW, Langeberg LK, Huganir RL, and Scott JD (2002). Regulation of GluR1 by the A-kinase anchoring protein 79 (AKAP79) signaling complex shares properties with long-term depression. J. Neurosci 22, 3044–3051. 11943807 10.1523/JNEUROSCI.22-08-03044.2002 PMC6757527

[17] Woolfrey KM, and Dell’Acqua ML (2015). Coordination of Protein Phosphorylation and Dephosphorylation in Synaptic Plasticity. J. Biol. Chem 290, 28604–28612. 26453308 10.1074/jbc.R115.657262 PMC4661375

[18] Roche KW, O’Brien RJ, Mammen AL, Bernhardt J, and Huganir RL (1996). Characterization of multiple phosphorylation sites on the AMPA receptor GluR1 subunit. Neuron 16, 1179–1188. 8663994 10.1016/s0896-6273(00)80144-0

[19] Lee HK, Barbarosie M, Kameyama K, Bear MF, and Huganir RL (2000). Regulation of distinct AMPA receptor phosphorylation sites during bidirectional synaptic plasticity. Nature 405, 955–959. 10879537 10.1038/35016089

[20] Lee HK, Takamiya K, He K, Song L, and Huganir RL (2010). Specific roles of AMPA receptor subunit GluR1 (GluA1) phosphorylation sites in regulating synaptic plasticity in the CA1 region of hippocampus. J. Neurophysiol 103, 479–489. 19906877 10.1152/jn.00835.2009 PMC2807233

[21] Esteban JA, Shi SH, Wilson C, Nuriya M, Huganir RL, and Malinow R (2003). PKA phosphorylation of AMPA receptor subunits controls synaptic trafficking underlying plasticity. Nat. Neurosci 6, 136–143. 12536214 10.1038/nn997

[22] Ehlers MD (2000). Reinsertion or degradation of AMPA receptors determined by activity-dependent endocytic sorting. Neuron 28, 511–525. 11144360 10.1016/s0896-6273(00)00129-x

[23] Sun X, Zhao Y, and Wolf ME (2005). Dopamine receptor stimulation modulates AMPA receptor synaptic insertion in prefrontal cortex neurons. J. Neurosci 25, 7342–7351. 16093384 10.1523/JNEUROSCI.4603-04.2005 PMC6725299

[24] Swayze RD, Lisé MF, Levinson JN, Phillips A, and El-Husseini A (2004). Modulation of dopamine mediated phosphorylation of AMPA receptors by PSD-95 and AKAP79/150. Neuropharmacology 47, 764–778. 15458848 10.1016/j.neuropharm.2004.07.014

[25] Oh MC, Derkach VA, Guire ES, and Soderling TR (2006). Extrasynaptic Membrane Trafficking Regulated by GluR1 Serine 845 Phosphorylation Primes AMPA Receptors for Long-term Potentiation. J. Biol. Chem 281, 752–758. 16272153 10.1074/jbc.M509677200

[26] Man H-Y, Sekine-Aizawa Y, and Huganir RL (2007). Regulation of {alpha}-amino-3-hydroxy-5-methyl-4-isoxazolepropionic acid receptor trafficking through PKA phosphorylation of the Glu receptor 1 subunit. Proc. Natl. Acad. Sci. USA 104, 3579–3584. 17360685 10.1073/pnas.0611698104 PMC1805611

[27] He K, Song L, Cummings LW, Goldman J, Huganir RL, and Lee HK (2009). Stabilization of Ca2+-permeable AMPA receptors at perisynaptic sites by GluR1-S845 phosphorylation. Proc. Natl. Acad. Sci. USA 106, 20033–20038. 19892736 10.1073/pnas.0910338106 PMC2785287

[28] Lee HK, Takamiya K, Han JS, Man H, Kim CH, Rumbaugh G, Yu S, Ding L, He C, Petralia RS, (2003). Phosphorylation of the AMPA receptor GluR1 subunit is required for synaptic plasticity and retention of spatial memory. Cell 112, 631–643. 12628184 10.1016/s0092-8674(03)00122-3

[29] Seol GH, Ziburkus J, Huang S, Song L, Kim IT, Takamiya K, Huganir RL, Lee HK, and Kirkwood A (2007). Neuromodulators control the polarity of spike-timing-dependent synaptic plasticity. Neuron 55, 919–929. 17880895 10.1016/j.neuron.2007.08.013 PMC2756178

[30] Park P, Sanderson TM, Amici M, Choi SL, Bortolotto ZA, Zhuo M, Kaang BK, and Collingridge GL (2016). Calcium-Permeable AMPA Receptors Mediate the Induction of the Protein Kinase A-Dependent Component of Long-Term Potentiation in the Hippocampus. J. Neurosci 36, 622–631. 26758849 10.1523/JNEUROSCI.3625-15.2016 PMC4710778

[31] Park P, Georgiou J, Sanderson TM, Ko KH, Kang H, Kim JI, Bradley CA, Bortolotto ZA, Zhuo M, Kaang BK, and Collingridge GL (2021). PKA drives an increase in AMPA receptor unitary conductance during LTP in the hippocampus. Nat. Commun 12, 413. 33462202 10.1038/s41467-020-20523-3 PMC7814032

[32] Qian H, Matt L, Zhang M, Nguyen M, Patriarchi T, Koval OM, Anderson ME, He K, Lee HK, and Hell JW (2012). β2-Adrenergic receptor supports prolonged theta tetanus-induced LTP. J. Neurophysiol 107, 2703–2712. 22338020 10.1152/jn.00374.2011 PMC3362273

[33] Bowen AB, Bourke AM, Hiester BG, Hanus C, and Kennedy MJ (2017). Golgi-independent secretory trafficking through recycling endosomes in neuronal dendrites and spines. eLife 6, e27362. 28875935 10.7554/eLife.27362 PMC5624785

[34] Yang Y, Wang XB, Frerking M, and Zhou Q (2008). Delivery of AMPA receptors to perisynaptic sites precedes the full expression of long-term potentiation. Proc. Natl. Acad. Sci. USA 105, 11388–11393. 18682558 10.1073/pnas.0802978105 PMC2496888

[35] Yang Y, Wang XB, Frerking M, and Zhou Q (2008). Spine expansion and stabilization associated with long-term potentiation. J. Neurosci 28, 5740–5751. 18509035 10.1523/JNEUROSCI.3998-07.2008 PMC2561912

[36] Yang Y, Wang XB, and Zhou Q (2010). Perisynaptic GluR2-lacking AMPA receptors control the reversibility of synaptic and spines modifications. Proc. Natl. Acad. Sci. USA 107, 11999–12004. 20547835 10.1073/pnas.0913004107 PMC2900706

[37] Opazo P, Labrecque S, Tigaret CM, Frouin A, Wiseman PW, De Koninck P, and Choquet D (2010). CaMKII triggers the diffusional trapping of surface AMPARs through phosphorylation of stargazin. Neuron 67, 239–252. 20670832 10.1016/j.neuron.2010.06.007

[38] Hafner AS, Penn AC, Grillo-Bosch D, Retailleau N, Poujol C, Philippat A, Coussen F, Sainlos M, Opazo P, and Choquet D (2015). Lengthening of the Stargazin Cytoplasmic Tail Increases Synaptic Transmission by Promoting Interaction to Deeper Domains of PSD-95. Neuron 86, 475–489. 25843401 10.1016/j.neuron.2015.03.013

[39] Penn AC, Zhang CL, Georges F, Royer L, Breillat C, Hosy E, Petersen JD, Humeau Y, and Choquet D (2017). Hippocampal LTP and contextual learning require surface diffusion of AMPA receptors. Nature 549, 384–388. 28902836 10.1038/nature23658 PMC5683353

[40] Thomas MJ, Moody TD, Makhinson M, and O’Dell TJ (1996). Activity-dependent beta-adrenergic modulation of low frequency stimulation induced LTP in the hippocampal CA1 region. Neuron 17, 475–482. 8816710 10.1016/s0896-6273(00)80179-8

[41] Gelinas JN, and Nguyen PV (2005). Beta-adrenergic receptor activation facilitates induction of a protein synthesis-dependent late phase of long-term potentiation. J. Neurosci 25, 3294–3303. 15800184 10.1523/JNEUROSCI.4175-04.2005 PMC6724894

[42] Lin YW, Min MY, Chiu TH, and Yang HW (2003). Enhancement of associative long-term potentiation by activation of beta-adrenergic receptors at CA1 synapses in rat hippocampal slices. J. Neurosci 23, 4173–4181. 12764105 10.1523/JNEUROSCI.23-10-04173.2003 PMC6741099

[43] Walling SG, and Harley CW (2004). Locus ceruleus activation initiates delayed synaptic potentiation of perforant path input to the dentate gyrus in awake rats: a novel beta-adrenergic- and protein synthesis-dependent mammalian plasticity mechanism. J. Neurosci 24, 598–604. 14736844 10.1523/JNEUROSCI.4426-03.2004 PMC6729256

[44] Katsuki H, Izumi Y, and Zorumski CF (1997). Noradrenergic regulation of synaptic plasticity in the hippocampal CA1 region. J. Neurophysiol 77, 3013–3020. 9212253 10.1152/jn.1997.77.6.3013

[45] Birnie MT, Claydon MDB, Troy O, Flynn BP, Yoshimura M, Kershaw YM, Zhao Z, Demski-Allen RCR, Barker GRI, Warburton EC, (2023). Circadian regulation of hippocampal function is disrupted with corticosteroid treatment. Proc. Natl. Acad. Sci. USA 120, e2211996120. 10.1073/pnas.2211996120 PMC10104554 37023133

[46] Patriarchi T, Qian H, Di Biase V, Malik ZA, Chowdhury D, Price JL, Hammes EA, Buonarati OR, Westenbroek RE, Catterall WA, (2016). Phosphorylation of Cav1.2 on S1928 Uncouples the L-type Ca2+ Channel from the β2 Adrenergic Receptor. EMBO J 35, 1330–1345. 27103070 10.15252/embj.201593409 PMC4910527

[47] Bock A, Annibale P, Konrad C, Hannawacker A, Anton SE, Maiellaro I, Zabel U, Sivaramakrishnan S, Falcke M, and Lohse MJ (2020). Optical Mapping of cAMP Signaling at the Nanometer Scale. Cell 182, 1519–1530.e17. 32846156 10.1016/j.cell.2020.07.035 PMC7984459

[48] Anton SE, Kayser C, Maiellaro I, Nemec K, Möller J, Koschinski A, Zaccolo M, Annibale P, Falcke M, Lohse MJ, and Bock A (2022). Receptor-associated independent cAMP nanodomains mediate spatiotemporal specificity of GPCR signaling. Cell 185, 1130–1142.e11. 35294858 10.1016/j.cell.2022.02.011

[49] Calebiro D, Nikolaev VO, Gagliani MC, de Filippis T, Dees C, Tacchetti C, Persani L, and Lohse MJ (2009). Persistent cAMP-signals triggered by internalized G-protein-coupled receptors. PLoS Biol. 7, e1000172. 10.1371/journal.pbio.1000172 PMC2718703 19688034

[50] Ferrandon S, Feinstein TN, Castro M, Wang B, Bouley R, Potts JT, Gardella TJ, and Vilardaga JP (2009). Sustained cyclic AMP production by parathyroid hormone receptor endocytosis. Nat. Chem. Biol 5, 734–742. 19701185 10.1038/nchembio.206 PMC3032084

[51] Feinstein TN, Yui N, Webber MJ, Wehbi VL, Stevenson HP, King JD Jr., Hallows KR, Brown D, Bouley R, and Vilardaga JP (2013). Noncanonical control of vasopressin receptor type 2 signaling by retromer and arrestin. J. Biol. Chem 288, 27849–27860. 23935101 10.1074/jbc.M112.445098 PMC3784700

[52] Irannejad R, Tomshine JC, Tomshine JR, Chevalier M, Mahoney JP, Steyaert J, Rasmussen SGF, Sunahara RK, El-Samad H, Huang B, and von Zastrow M (2013). Conformational biosensors reveal GPCR signalling from endosomes. Nature 495, 534–538. 23515162 10.1038/nature12000 PMC3835555

[53] Tsvetanova NG, and von Zastrow M (2014). Spatial encoding of cyclic AMP signaling specificity by GPCR endocytosis. Nat. Chem. Biol 10, 1061–1065. 25362359 10.1038/nchembio.1665 PMC4232470

[54] Koepsell H, Lips K, and Volk C (2007). Polyspecific organic cation transporters: structure, function, physiological roles, and biopharmaceutical implications. Pharm. Res 24, 1227–1251. 17473959 10.1007/s11095-007-9254-z

[55] Couroussé T, and Gautron S (2015). Role of organic cation transporters (OCTs) in the brain. Pharmacol. Ther 146, 94–103. 25251364 10.1016/j.pharmthera.2014.09.008

[56] Irannejad R, Pessino V, Mika D, Huang B, Wedegaertner PB, Conti M, and von Zastrow M (2017). Functional selectivity of GPCR-directed drug action through location bias. Nat. Chem. Biol 13, 799–806. 28553949 10.1038/nchembio.2389 PMC5733145

[57] Puri NM, Romano GR, Lin TY, Mai QN, and Irannejad R (2022). The organic cation transporter 2 regulates dopamine D1 receptor signaling at the Golgi apparatus. eLife 11, e75468. 35467530 10.7554/eLife.75468 PMC9098220

[58] Engel K, Zhou M, and Wang J (2004). Identification and characterization of a novel monoamine transporter in the human brain. J. Biol. Chem 279, 50042–50049. 15448143 10.1074/jbc.M407913200

[59] Gasser PJ, Orchinik M, Raju I, and Lowry CA (2009). Distribution of organic cation transporter 3, a corticosterone-sensitive monoamine transporter, in the rat brain. J. Comp. Neurol 512, 529–555. 19025979 10.1002/cne.21921

[60] Vialou V, Balasse L, Callebert J, Launay JM, Giros B, and Gautron S (2008). Altered aminergic neurotransmission in the brain of organic cation transporter 3-deficient mice. J. Neurochem 106, 1471–1482. 18513366 10.1111/j.1471-4159.2008.05506.x

[61] Bacq A, Balasse L, Biala G, Guiard B, Gardier AM, Schinkel A, Louis F, Vialou V, Martres MP, Chevarin C, (2012). Organic cation transporter 2 controls brain norepinephrine and serotonin clearance and antidepressant response. Mol. Psychiatry 17, 926–939. 21769100 10.1038/mp.2011.87

[62] Gilman TL, George CM, Vitela M, Herrera-Rosales M, Basiouny MS, Koek W, and Daws LC (2018). Constitutive plasma membrane monoamine transporter (PMAT, Slc29a4) deficiency subtly affects anxiety-like and coping behaviours. Eur. J. Neurosci10.1111/ejn.13968 PMC6252160 29797618

[63] Hill JE, Makky K, Shrestha L, Hillard CJ, and Gasser PJ (2011). Natural and synthetic corticosteroids inhibit uptake 2-mediated transport in CNS neurons. Physiol. Behav 104, 306–311. 21081135 10.1016/j.physbeh.2010.11.012

[64] Shang T, Uihlein AV, Van Asten J, Kalyanaraman B, and Hillard CJ (2003). 1-Methyl-4-phenylpyridinium accumulates in cerebellar granule neurons via organic cation transporter 3. J. Neurochem 85, 358–367. 12675912 10.1046/j.1471-4159.2003.01686.x

[65] Gründemann D, Schechinger B, Rappold GA, and Schomig E (1998). Molecular identification of the corticosterone-sensitive extraneuronal catecholamine transporter. Nat. Neurosci 1, 349–351. 10196521 10.1038/1557

[66] Hayer-Zillgen M, Brüss M, and Bönisch H (2002). Expression and pharmacological profile of the human organic cation transporters hOCT1, hOCT2 and hOCT3. Br. J. Pharmacol 136, 829–836. 12110607 10.1038/sj.bjp.0704785 PMC1573414

[67] Engel K, and Wang J (2005). Interaction of organic cations with a newly identified plasma membrane monoamine transporter. Mol. Pharmacol 68, 1397–1407. 16099839 10.1124/mol.105.016832

[68] Fraser-Spears R, Krause-Heuer AM, Basiouny M, Mayer FP, Manishimwe R, Wyatt NA, Dobrowolski JC, Roberts MP, Greguric I, Kumar N, (2019). Comparative analysis of novel decynium-22 analogs to inhibit transport by the low-affinity, high-capacity monoamine transporters, organic cation transporters 2 and 3, and plasma membrane monoamine transporter. Eur. J. Pharmacol 842, 351–364. 30473490 10.1016/j.ejphar.2018.10.028 PMC6440482

[69] Koepsell H (2021). General Overview of Organic Cation Transporters in Brain. Handb. Exp. Pharmacol 266, 1–39. 33782773 10.1007/164_2021_449

[70] Duan H, Hu T, Foti RS, Pan Y, Swaan PW, and Wang J (2015). Potent and Selective Inhibition of Plasma Membrane Monoamine Transporter by HIV Protease Inhibitors. Drug Metab. Dispos 43, 1773–1780. 26285765 10.1124/dmd.115.064824 PMC4613949

[71] Mammen AL, Kameyama K, Roche KW, and Huganir RL (1997). Phosphorylation of the alpha-amino-3-hydroxy-5-methylisoxazole4-propionic acid receptor GluR1 subunit by calcium/calmodulin-dependent kinase II. J. Biol. Chem 272, 32528–32533. 9405465 10.1074/jbc.272.51.32528

[72] Torres GE, Gainetdinov RR, and Caron MG (2003). Plasma membrane monoamine transporters: structure, regulation and function. Nat. Rev. Neurosci 4, 13–25. 12511858 10.1038/nrn1008

[73] Qian H, Patriarchi T, Price JL, Matt L, Lee B, Nieves-Cintrón M, Buonarati OR, Chowdhury D, Nanou E, Nystoriak MA, (2017). Phosphorylation of Ser1928 mediates the enhanced activity of the L-type Ca2+ channel Cav1.2 by the β2-adrenergic receptor in neurons. Sci. Signal 10, eaaf9659. 10.1126/scisignal.aaf9659 PMC5310946 28119465

[74] Lipinski CA (2000). Drug-like properties and the causes of poor solubility and poor permeability. J. Pharmacol. Toxicol. Methods 44, 235–249. 11274893 10.1016/s1056-8719(00)00107-6

[75] Soliman K, Grimm F, Wurm CA, and Egner A (2021). Predicting the membrane permeability of organic fluorescent probes by the deep neural network based lipophilicity descriptor DeepFl-LogP. Sci. Rep 11, 6991. 33772099 10.1038/s41598-021-86460-3 PMC7997998

[76] Lu Y, Allen M, Halt AR, Weisenhaus M, Dallapiazza RF, Hall DD, Usachev YM, McKnight GS, and Hell JW (2007). Age-dependent requirement of AKAP150-anchored PKA and GluR2-lacking AMPA receptors in LTP. EMBO J. 26, 4879–4890. 17972919 10.1038/sj.emboj.7601884 PMC2099463

[77] Sanderson JL, Freund RK, Gorski JA, and Dell’Acqua ML (2021). β-Amyloid disruption of LTP/LTD balance is mediated by AKAP150-anchored PKA and Calcineurin regulation of Ca^2+^-permeable AMPA receptors. Cell Rep. 37, 109786. 10.1016/j.celrep.2021.109786 PMC8530450 34610314

[78] Granger AJ, Shi Y, Lu W, Cerpas M, and Nicoll RA (2013). LTP requires a reserve pool of glutamate receptors independent of subunit type. Nature 493, 495–500. 23235828 10.1038/nature11775 PMC3998843

[79] Mizuseki K, Sirota A, Pastalkova E, and Buzsáki G (2009). Theta oscillations provide temporal windows for local circuit computation in the entorhinal-hippocampal loop. Neuron 64, 267–280. 19874793 10.1016/j.neuron.2009.08.037 PMC2771122

[80] Mizuseki K, Diba K, Pastalkova E, and Buzsáki G (2011). Hippocampal CA1 pyramidal cells form functionally distinct sublayers. Nat. Neurosci 14, 1174–1181. 21822270 10.1038/nn.2894 PMC3164922

[81] Vyazovskiy VV, Cirelli C, Pfister-Genskow M, Faraguna U, and Tononi G (2008). Molecular and electrophysiological evidence for net synaptic potentiation in wake and depression in sleep. Nat. Neurosci 11, 200–208. 18204445 10.1038/nn2035

[82] Diering GH, Nirujogi RS, Roth RH, Worley PF, Pandey A, and Huganir RL (2017). Homer1a drives homeostatic scaling-down of excitatory synapses during sleep. Science 355, 511–515. 28154077 10.1126/science.aai8355 PMC5382711

[83] Friedman AH, and Walker CA (1968). Circadian rhythms in rat mid-brain and caudate nucleus biogenic amine levels. J. Physiol 197, 77–85. 5675092 10.1113/jphysiol.1968.sp008547 PMC1351786

[84] Manshardt J, and Wurtman RJ (1968). Daily rhythm in the noradrenaline content of rat hypothalamus. Nature 217, 574–575. 5641116 10.1038/217574a0

[85] Aston-Jones G, and Bloom FE (1981). Norepinephrine-containing locus coeruleus neurons in behaving rats exhibit pronounced responses to non-noxious environmental stimuli. J. Neurosci 1, 887–900. 7346593 10.1523/JNEUROSCI.01-08-00887.1981 PMC6564231

[86] Aston-Jones G, and Bloom FE (1981). Activity of norepinephrine-containing locus coeruleus neurons in behaving rats anticipates fluctuations in the sleep-waking cycle. J. Neurosci 1, 876–886. 7346592 10.1523/JNEUROSCI.01-08-00876.1981 PMC6564235

[87] Aston-Jones G, Chen S, Zhu Y, and Oshinsky ML (2001). A neural circuit for circadian regulation of arousal. Nat. Neurosci 4, 732–738. 11426230 10.1038/89522

[88] Berridge CW, and Waterhouse BD (2003). The locus coeruleus-noradrenergic system: modulation of behavioral state and state-dependent cognitive processes. Brain Res. Brain Res. Rev 42, 33–84. 12668290 10.1016/s0165-0173(03)00143-7

[89] Leach S, and Suzuki K (2020). Adrenergic Signaling in Circadian Control of Immunity. Front. Immunol 11, 1235. 32714319 10.3389/fimmu.2020.01235 PMC7344327

[90] Stoddart LA, Kilpatrick LE, Briddon SJ, and Hill SJ (2015). Probing the pharmacology of G protein-coupled receptors with fluorescent ligands. Neuropharmacology 98, 48–57. 25979488 10.1016/j.neuropharm.2015.04.033

[91] Bowman SL, Shiwarski DJ, and Puthenveedu MA (2016). Distinct G protein-coupled receptor recycling pathways allow spatial control of downstream G protein signaling. J. Cell Biol 214, 797–806. 27646272 10.1083/jcb.201512068 PMC5037407

[92] Tian X, Irannejad R, Bowman SL, Du Y, Puthenveedu MA, von Zastrow M, and Benovic JL (2016). The α-Arrestin ARRDC3 Regulates the Endosomal Residence Time and Intracellular Signaling of the β2-Adrenergic Receptor. J. Biol. Chem 291, 14510–14525. 27226565 10.1074/jbc.M116.716589 PMC4938174

[93] Hiester BG, Bourke AM, Sinnen BL, Cook SG, Gibson ES, Smith KR, and Kennedy MJ (2017). L-Type Voltage-Gated Ca^2+^ Channels Regulate Synaptic-Activity-Triggered Recycling Endosome Fusion in Neuronal Dendrites. Cell Rep. 21, 2134–2146. 29166605 10.1016/j.celrep.2017.10.105 PMC6466620

[94] Shen A, Nieves-Cintron M, Deng Y, Shi Q, Chowdhury D, Qi J, Hell JW, Navedo MF, and Xiang YK (2018). Functionally distinct and selectively phosphorylated GPCR subpopulations co-exist in a single cell. Nat. Commun 9, 1050. 29535304 10.1038/s41467-018-03459-7 PMC5849717

[95] Zhang L, Zhang P, Wang G, Zhang H, Zhang Y, Yu Y, Zhang M, Xiao J, Crespo P, Hell JW, (2018). Ras and Rap Signal Bidirectional Synaptic Plasticity via Distinct Subcellular Microdomains. Neuron 98, 783–800.e4. 29706584 10.1016/j.neuron.2018.03.049 PMC6192044

[96] Zwart R, Verhaagh S, Buitelaar M, Popp-Snijders C, and Barlow DP (2001). Impaired activity of the extraneuronal monoamine transporter system known as uptake-2 in Orct3/Slc22a3-deficient mice. Mol. Cell Biol 21, 4188–4196. 11390648 10.1128/MCB.21.13.4188-4196.2001 PMC87080

[97] Duan H, and Wang J (2013). Impaired monoamine and organic cation uptake in choroid plexus in mice with targeted disruption of the plasma membrane monoamine transporter (Slc29a4) gene. J. Biol. Chem 288, 3535–3544. 23255610 10.1074/jbc.M112.436972 PMC3561572

[98] Davare MA, Dong F, Rubin CS, and Hell JW (1999). The A-kinase anchor protein MAP2B and cAMP-dependent protein kinase are associated with class C L-type calcium channels in neurons. J. Biol. Chem 274, 30280–30287. 10514522 10.1074/jbc.274.42.30280

[99] Hall DD, Feekes JA, Arachchige Don AS, Shi M, Hamid J, Chen L, Strack S, Zamponi GW, Horne MC, and Hell JW (2006). Binding of protein phosphatase 2A to the L-type calcium channel Cav1.2 next to Ser1928, its main PKA site, is critical for Ser1928 dephosphorylation. Biochemistry 45, 3448–3459. 16519540 10.1021/bi051593z

[100] Hall DD, Davare MA, Shi M, Allen ML, Weisenhaus M, McKnight GS, and Hell JW (2007). Critical role of cAMP-dependent protein kinase anchoring to the L-type calcium channel Cav1.2 via A-kinase anchor protein 150 in neurons. Biochemistry 46, 1635–1646. 17279627 10.1021/bi062217x

[101] Davare MA, and Hell JW (2003). Increased phosphorylation of the neuronal L-type Ca(2+) channel Ca(v)1.2 during aging. Proc. Natl. Acad. Sci 100, 16018–16023. 14665691 10.1073/pnas.2236970100 PMC307685

[102] Matt L, Kim K, Hergarden AC, Patriarchi T, Malik ZA, Park DK, Chowdhury D, Buonarati OR, Henderson PB, Gökçek Saraç Ç, (2018). α-Actinin Anchors PSD-95 at Postsynaptic Sites. Neuron 97, 1094–1109.e9. 29429936 10.1016/j.neuron.2018.01.036 PMC5963734

