# Supplementary material for: Signaling by intracellular β2-adrenergic receptors regulates AMPA receptor trafficking and synaptic plasticity
Source: Cell Rep. Author manuscript; Available in PMC 2025 Nov 1. (PMC12579434; doi:10.1016/j.celrep.2025.116011)
Supplement: 1 [file NIHMS2107244-supplement-1.pdf]

Cell Reports, Volume *44*

Supplemental information

Signaling by intracellular β_2-adrenergic receptors regulates AMPA receptor trafficking and synaptic plasticity

Boram Lee, Xiaomin Xing, Erik A. Hammes, Zhuoer Zeng, Zoila M. Estrada-Tobar, Karam Kim, Kyle E. Ireton, Kwun Nok Mimi Man, Ariel A. Jacobi, Ruben A. Berumen, Justin C. Weiner, Ao Shen, Bing Xu, Joanne Wang, Paul J. Gasser, Manuel F. Navedo, Yang K. Xiang, Elva Díaz, Chao-Yin Chen, Mary C. Horne, and Johannes W. Hell

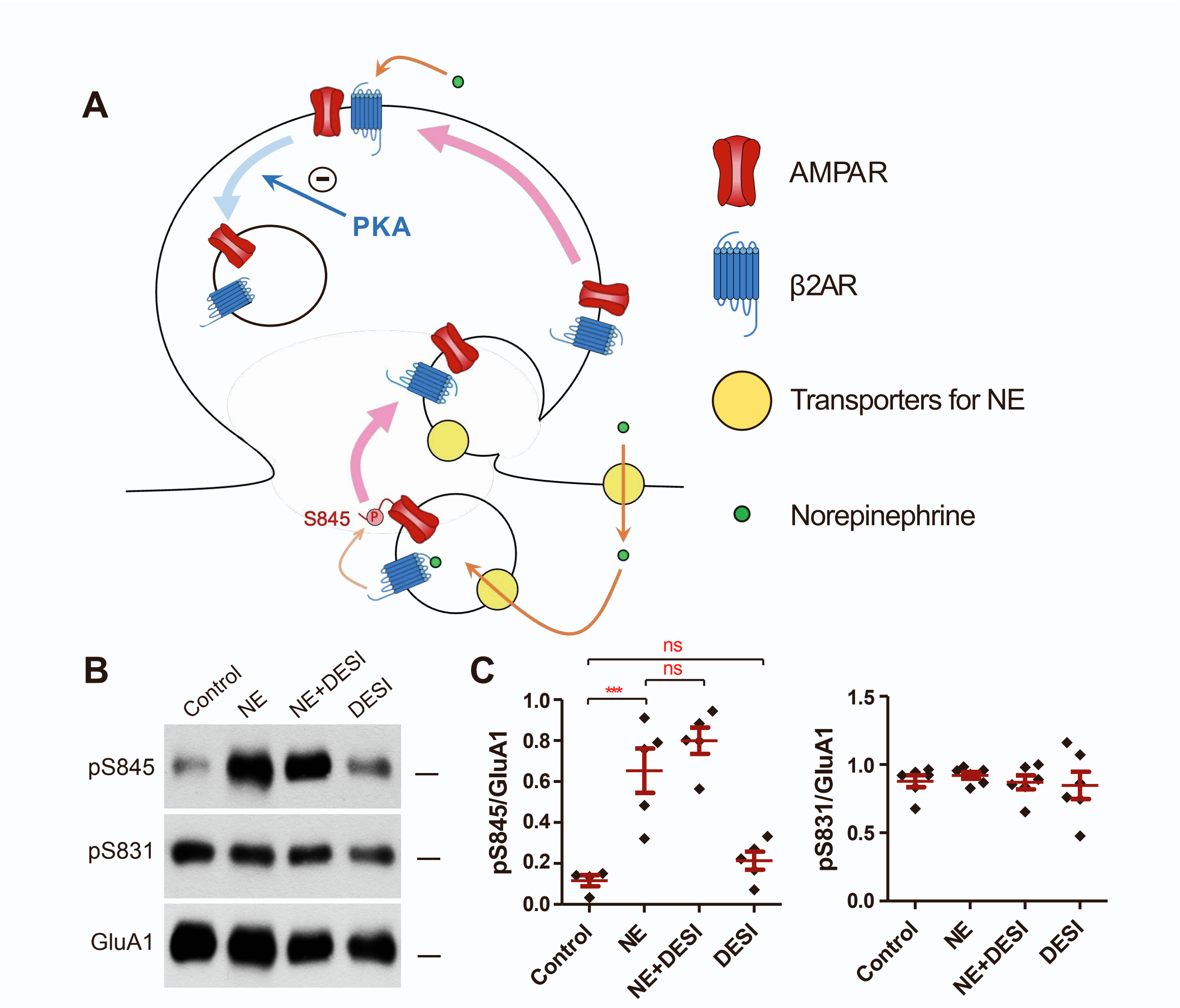

Supplemental Figure 1. Model of intracellular signaling by NE and effects of desipramine on GluA1 phosphorylation

(A) Model of intracellular signaling by NE. NE can act on β_2 AR at the cell surface and, after transport through the plasma membrane and vesicular membrane, on vesicular β_2 ARs to stimulate phosphorylation of S845 and thereby forward trafficking of AMPARs to the cell surface. At the same time, PKA activity in general inhibits endocytosis of AMPARs [1], which could augment the increase in surface GluA1 in conjunction with the increase in forward trafficking.

(B,C) Forebrain slices from WT mice were pre-incubated with ACSF for 5 min containing vehicle (H_2O) or 1 µM desipramine (DESI) before addition of vehicle (H_2O) or 1µM NE for 10 min, solubilization, ultracentrifugation, immunoprecipitation of GluA1, and sequential immunoblotting with antibodies against pS845, pS831, and the GluA1 C-terminus. For quantification, signals for pS845 and pS831 were normalized to signals for total GluA1 (C: n=5 from 5 mice; bars and whiskers represent means±SEM; data were analyzed by one-way ANOVA and significance of differences between control vs NE, NE vs NE+DESI, and control vs DESI by Fisher's least significant difference (LSD) post-hoc test (C); ***p<0.001, ns: not significant). The NET inhibitor DESI did not inhibit NE-induced S845 phosphorylation at all. Bars on right sides of each blot indicate the 100 kD marker position.

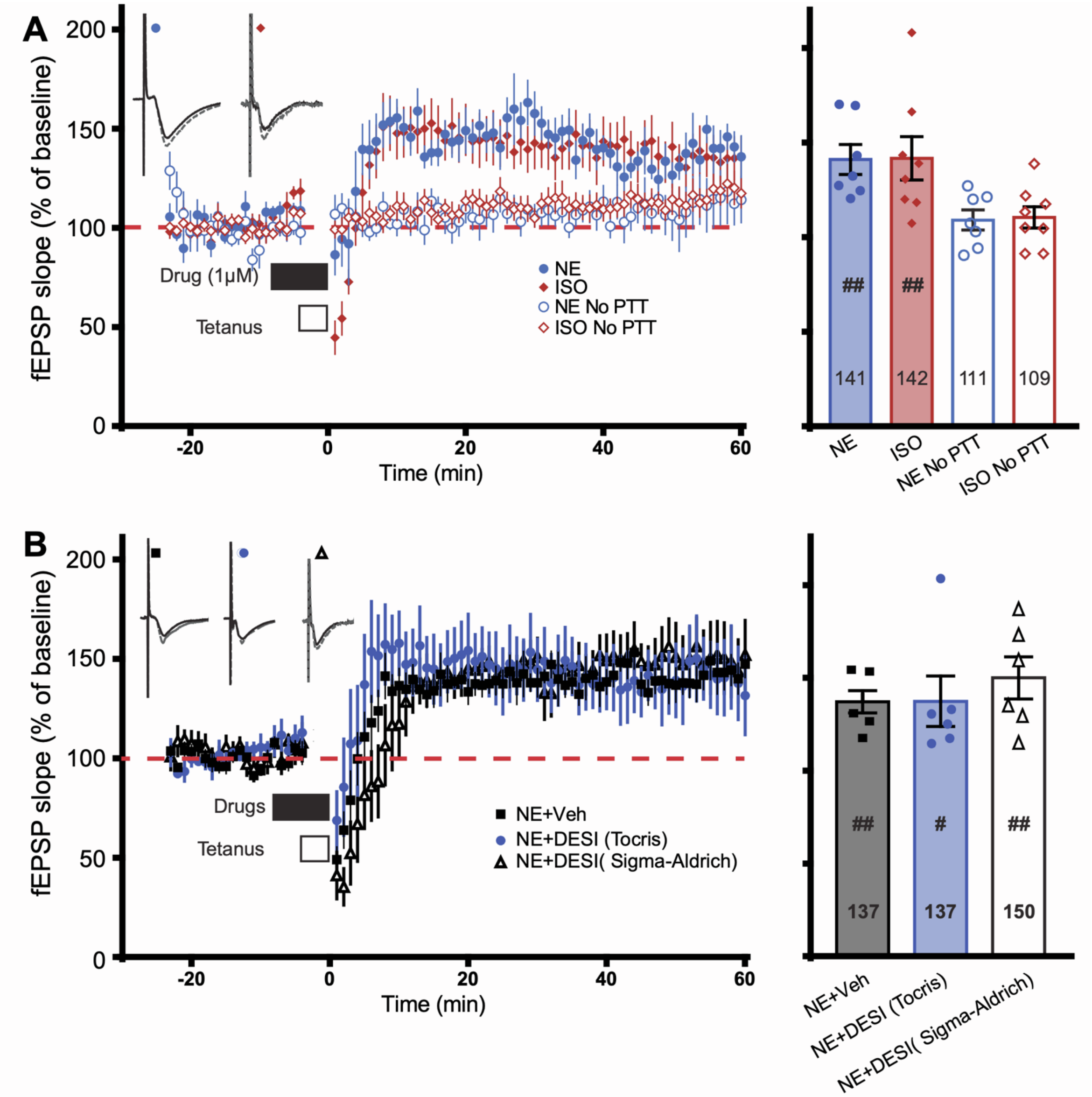

Supplemental Figure 2. NE and ISO minimally increased basal fEPSPs but permitted induction of PTT-LTP that is not sensitive to the NET inhibitor desipramine (DESI)
Field EPSPs were recorded from hippocampal slices before and after 5 Hz/3 min tetani (PTT; open horizontal bar). Plots show time course of initial slopes. The solid horizontal black bar indicates time of perfusion with 1 μM NE, 1 μM ISO, or NE+DESI (1 μM; 0.01% DMSO final concentration) from either Tocris or Sigma. Drug identity and integrity was confirmed by NMR spectroscopy. *Inserts:* Sample traces of fEPSP recordings ~20 min prior to (solid lines) and ~30 min post drug perfusion paired with tetanus (dashed lines). Bar graphs show level of fEPSPs 45-50 min after drug perfusion versus baseline (mean ± SEM; exact values inside bars). Paired t-test was used to determine significant difference of fEPSP slope averages from 5-10 min prior to and 45-50 min post drug treatment ($^{\#}p<0.05$, $^{\#\#}p<0.01$ versus before treatments equaling 100%).
(A) NE and ISO significantly increased synaptic transmission only when a PTT was given (filled symbols and bars) but not without a PTT (open symbols and bars; blue: NE; red: ISO).
(B) DESI did not inhibit PTT-LTP. One-way ANOVA was used to determine potential differences between treatment groups, without yielding any significance. Comparison of control versus combined DESI data by two-tailed t-test also did not show any significant inhibition by DESI.

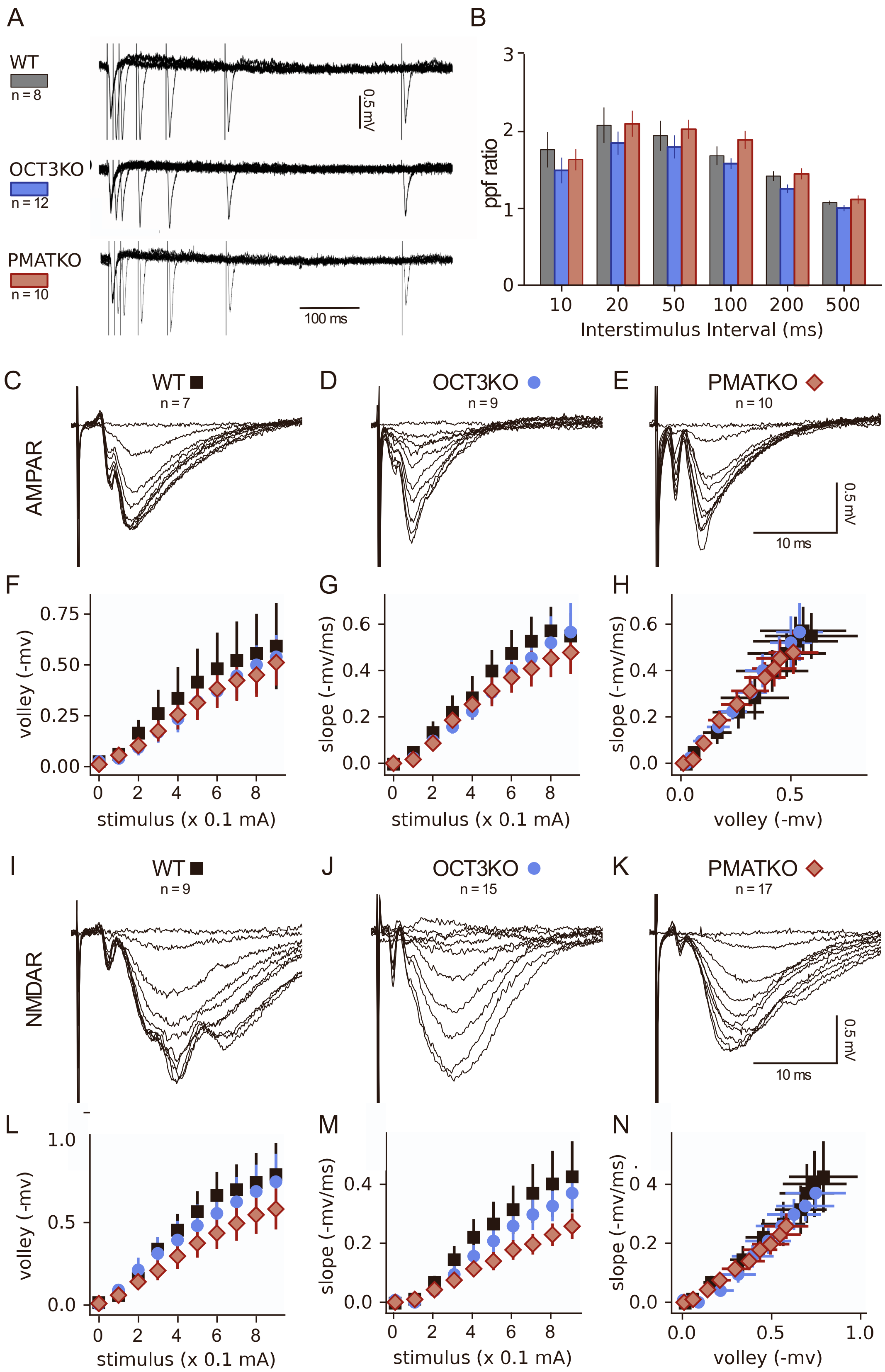

Supplemental Figure 3

Supplemental Figure 3. Synaptic transmission is unaltered in OCT3 and PMAT KO mice. Schaffer-collateral fEPSPs were recorded from acute hippocampal slices from 8-12 week-old wild-type (WT), OCT3 and PMAT KO mice.

(A,B) Paired-pulse facilitation (PPF) was not different for WT, OCT3 KO, and PMAT KO mice.

(A) Overlay of sample traces from representative recordings.

(B) Averages of PPF for WT (n = 8), OCT3 KO (n = 12), and PMAT KO (n = 10).

(C-H) Input-output relationships tested with increasing stimulus strength were not different between WT, OCT3 KO, and PMAT KO mice under standard conditions.

(C-E) Overlay of sample traces of fiber volleys followed by fEPSPs elicited by increasing stimulus strengths in slice from WT, OCT3 KO, and PMAT KO mice under standard conditions.

(F-H) Averages of presynaptic fiber volleys (F) and fEPSP slopes (G) were plotted against stimulus strengths and of fEPSP slopes against fiber volley amplitudes (H). No difference was observed between WT (n = 7), OCT3 KO (n = 9), and PMAT KO mice (n = 10).

(I-N) Input-output relationships tested with increasing stimulus strength were not different between WT, OCT3 KO, and PMAT KO mice with AMPARs blocked by NBQX and NMADRs unblocked by removal of Mg^{2+}.

(I-K) Sample traces of fiber volleys followed by fEPSPs elicited by increasing stimulus strengths in slice from WT, OCT3 KO, and PMAT KO mice with NBQX present and Mg^{2+} absent.

(L-N) Averages of presynaptic fiber volleys (L) and fEPSP slopes (M) were plotted against stimulus strengths and of fEPSP slopes against fiber volley amplitudes (N). No difference was observed between WT (n = 9), OCT3 KO (n = 15), and PMAT KO mice (n = 17).

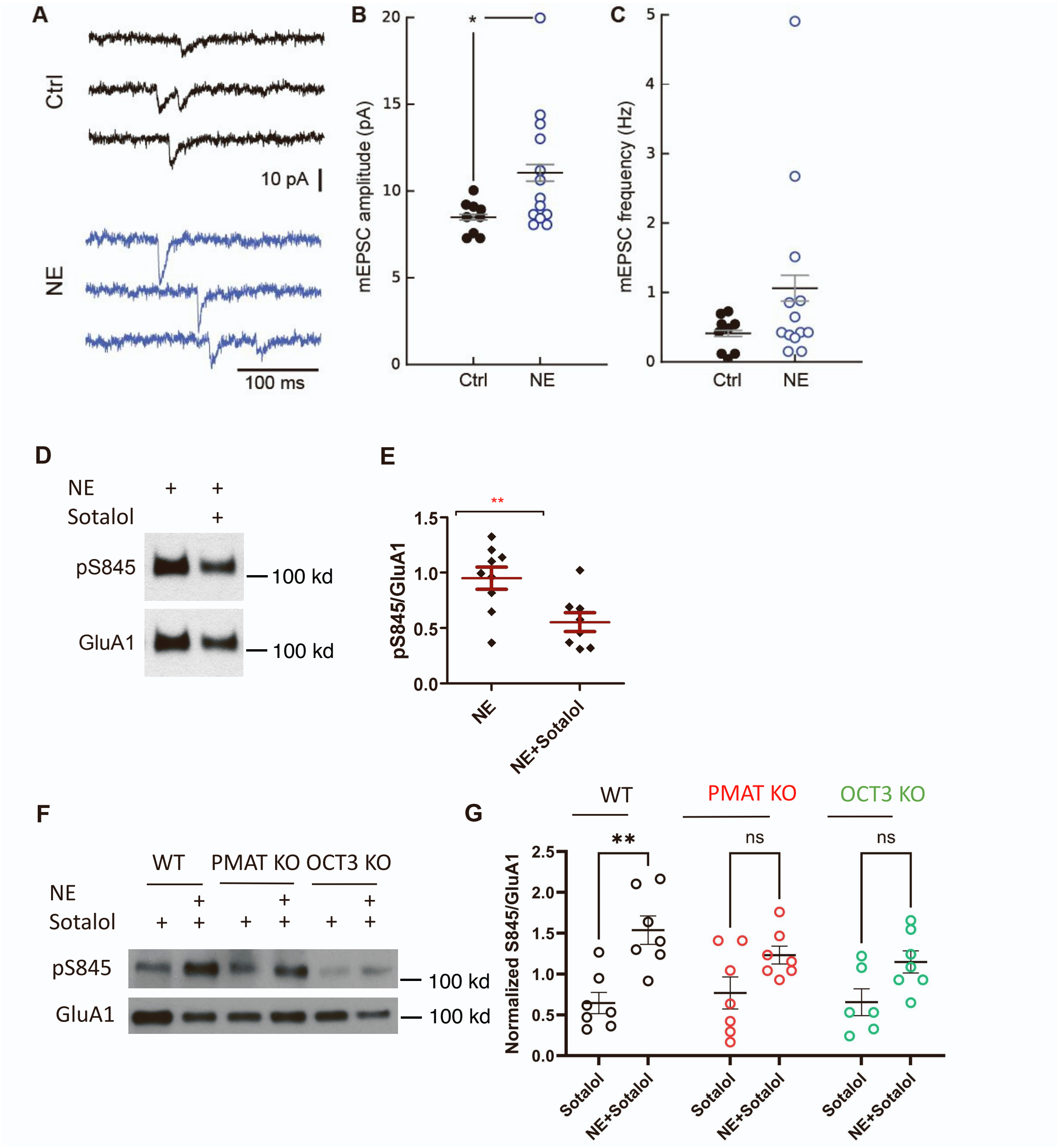

Supplemental Figure 4. Use of the membrane-impermeant β blocker sotalol to exclude contribution of β ARs at the cell surface.

(A) Representative mEPSC recordings from CA1 pyramidal neurons in slices from 13-25 day-old WT mice without (top 3 traces) and with (bottom 3 traces) 20 μM NE in the recording electrode.

(B,C) Averages of mEPSC amplitudes (B) and frequencies (C). NE increased mEPSC amplitude (*p < 0.05, t-test) despite the continuous presence of a saturating concentration of sotalol.

(D-G) Forebrain slices were pre-incubated with ACSF for 5 min containing vehicle (H_2O) or 100 μM sotalol before addition of 1μM NE for 10 min, immunoprecipitation of GluA1, and sequential immunoblotting with antibodies against pS845 and the GluA1 C-terminus.

(D,E) In WT slices, sotalol significantly reduced pS845 levels in the presence of NE, demonstrating its efficacy in inhibiting β_2 ARs at the cell surface (E: means±SEM; **p<0.01;t-test).

(F,G) In the presence of sotalol to block signaling from β_2 ARs at the cell surface NE still significantly increased phosphorylation of GluA1 on S845 in slices from WT mice (p=0.027) but from PMAT KO mice (p=0.287) or OCT3 KO mice (p=0.264; G: means±SEM; **p<0.01;t-test).

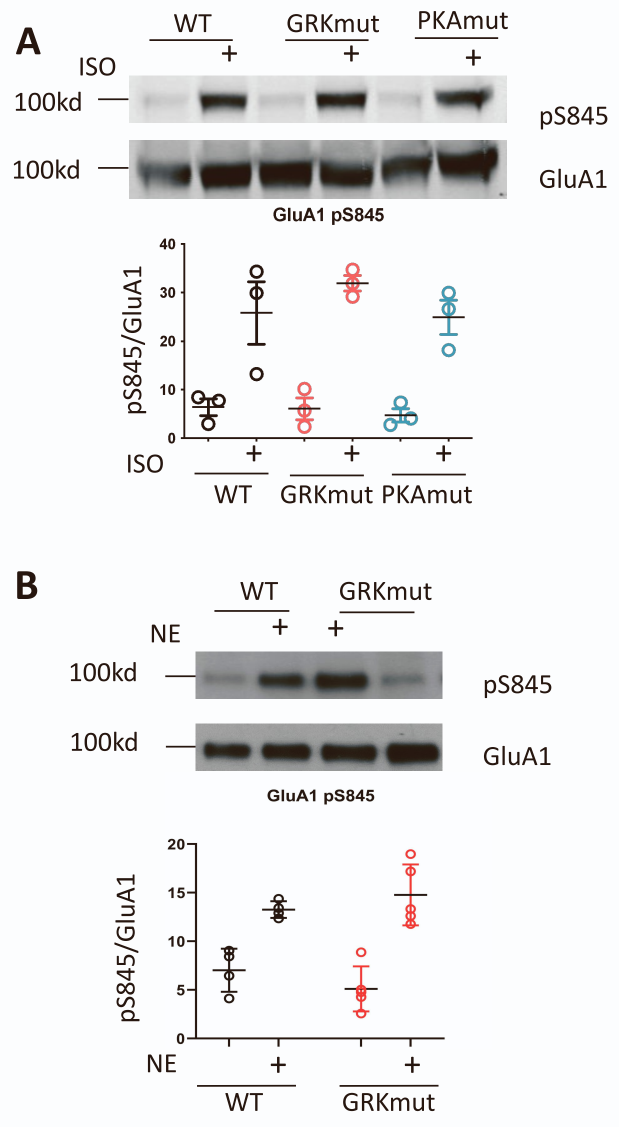

Supplemental Figure 5. ISO- and NE-induced S845 phosphorylation is not reduced in neurons with endocytosis-deficient β_2 ARs

(A) Hippocampal cultures from β_1/β_2 AR DKO mice were infected at 8 DIV with lentivirus (20 µl per 35 mm culture dish) for expression of WT β_2 AR or β_2 AR with point mutations at the G protein coupled receptor kinase (GRK) phosphorylation sites (S355A/S356A), rendering it endocytosis deficient or PKA phosphorylation sites (S261A/S262A), which does not affect endocytosis [2]. Cells were treated at 11 DIV with 1 µM ISO or vehicle (H_2O) for 5 mins and solubilized (1% Triton X-100) before ultracentrifugation and sequential immunoblotting of the supernatant with antibodies against pS845 and the GluA1 C-terminus (total GluA1).

(B) Acute forebrain slices from WT mice or S355A/S356A double-knock in (DKI) mice with point mutations at these GRK phosphorylation sites to render it endocytosis deficient were treated with 1 µM NE or vehicle (H_2O) for 5 mins and solubilized (1% Triton X-100) before ultracentrifugation, IP of GluA1 and sequential immunoblotting with antibodies against pS845 and the GluA1 C-terminus (total GluA1).

Dot plots represent the mean ± SEM of neurons from 3 (A) or 5 (B) independent experiments. There was no statistically significant difference in ISO-induced S845 phosphorylation between WT and any of the mutant β_2 ARs in either set of experiments (t-test).

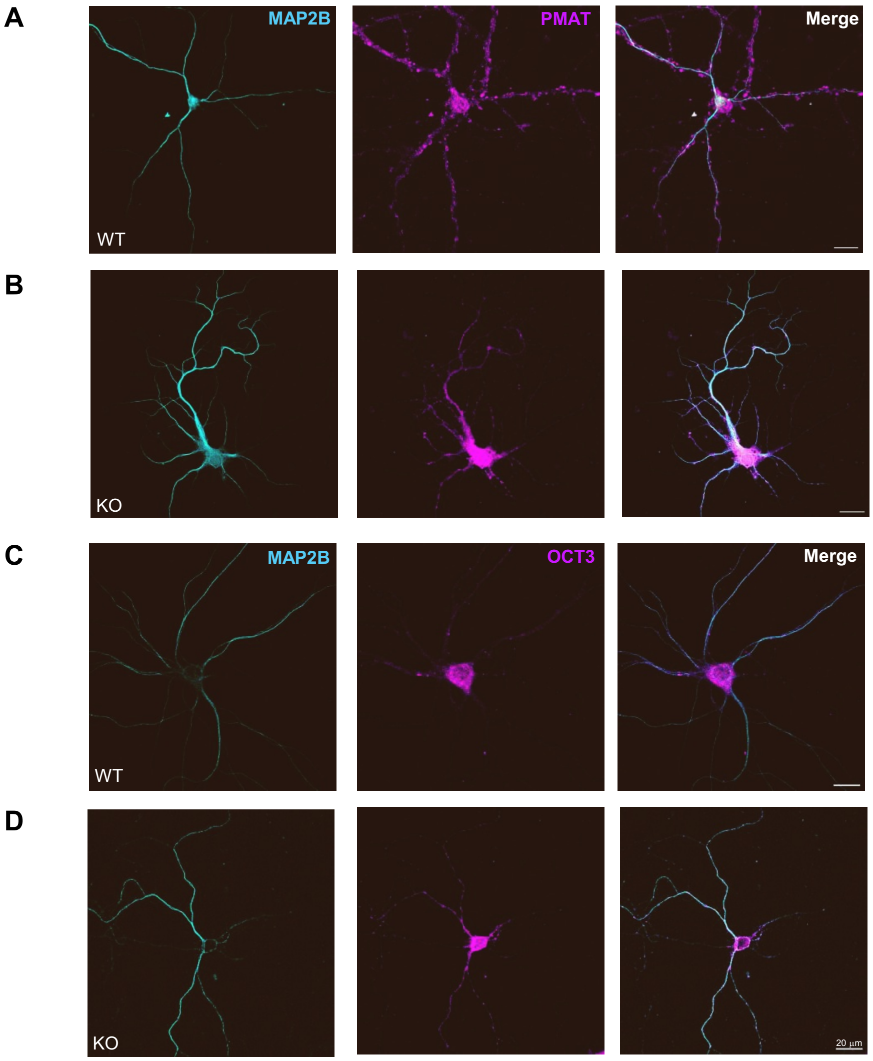

Supplemental Figure 6. Currently available PMAT and OCT3 antibodies show prominent and apparently non-specific staining in respective KO neurons
Hippocampal cultures from WT or respective KO neurons were fixed, permeabilized, and labeled at 15 DIV with antibodies against PMAT (A,B; magenta; antibody from Alamone at 1:100 dilution) or OCT3 (C,D; magenta; antibody from Novus Biologics at 1:100 dilution) plus MAP2B (cyan). Stainings with PMAT and OCT3 were comparable for WT (A,C) and respective KO neurons (B,D), suggesting non-specific staining. Scale bars: 20 µm.

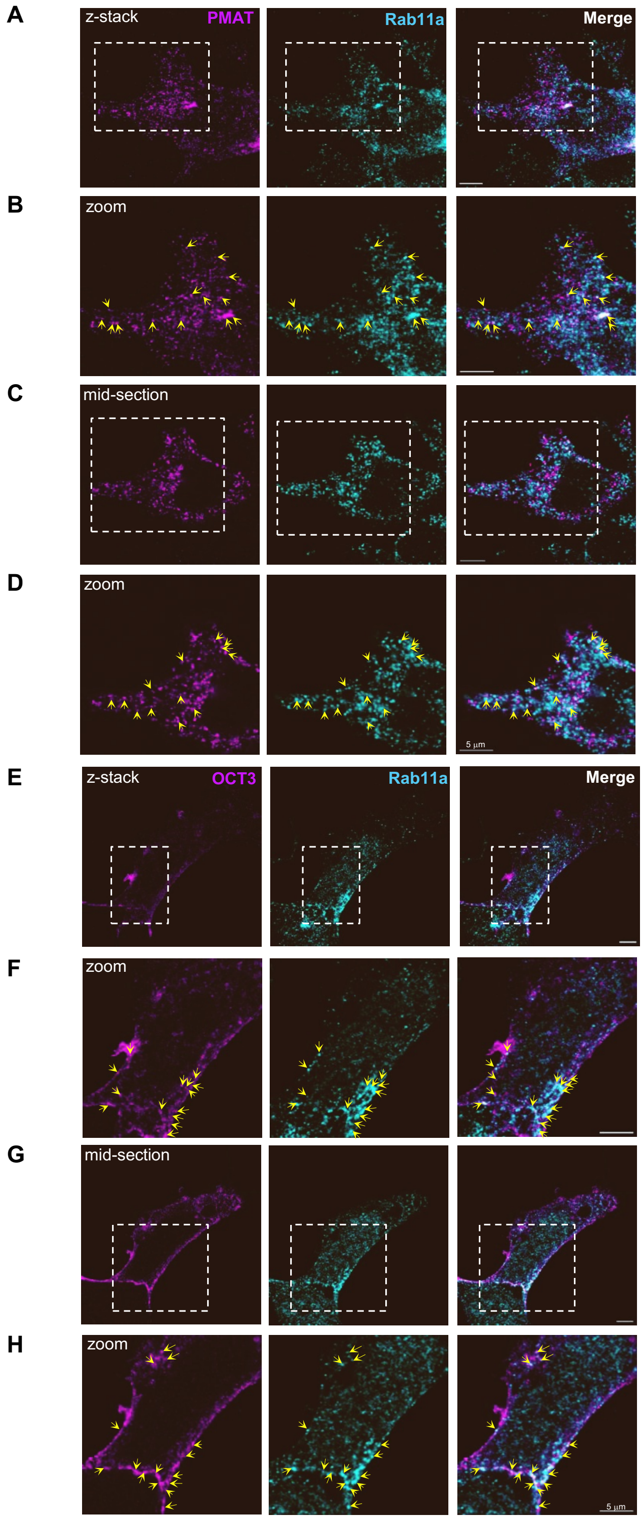

Supplemental Figure 7. Localization of eGFP-PMAT and eGFP-OCT3 in HEK293 cells
HEK293 cells were transfected with eGFP-PMAT (A-D, magenta) or eGFP-OCT3 (E-H, magenta). After 24 h cells were fixed, permeabilized, and labeled with an antibody against Rab11a (cyan). Shown are full z stack projections (A,B,E,F) and Individual middle-level z sections (C,D,G,H). Scale bars: 5 µm. Yellow arrows show puncta that colocalize for the transporter with Rab11a.

REFERENCES

1. Ehlers, M.D. (2000). Reinsertion or degradation of AMPA receptors determined by activity-dependent endocytic sorting. *Neuron 28*: 511-525.
2. Shen, A., Nieves-Cintron, M., Deng, Y., Shi, Q., Chowdhury, D., Qi, J., Hell, J.W., Navedo, M.F., and Xiang, Y.K. (2018). Functionally distinct and selectively phosphorylated GPCR subpopulations co-exist in a single cell. *Nature Communications 9*: 1050. https://www.ncbi.nlm.nih.gov/pubmed/29535304
